# An intrinsically disordered linker controlling the formation and the stability of the bacterial flagellar hook

**DOI:** 10.1186/s12915-017-0438-7

**Published:** 2017-10-27

**Authors:** Clive S. Barker, Irina V. Meshcheryakova, Alla S. Kostyukova, Peter L. Freddolino, Fadel A. Samatey

**Affiliations:** 1 Trans-membrane Trafficking Unit, Okinawa Institute of Science and Technology Graduate University, 1919-1 Tancha, Onna, Kunigami, Okinawa, 904-0495 Japan; 2 Voiland School of Chemical Engineering and Bioengineering, Washington State University, Pullman, Washington USA; 3 Department of Biological Chemistry, University of Michigan Medical School, Ann Arbor, Michigan USA

**Keywords:** Intrinsically disordered peptide, Supra-molecular complex, Protein stability, Motility, Universal joint, Bacterial flagellum

## Abstract

**Background:**

In a macro-molecular complex, any minor change may prove detrimental. For a supra-molecular nano-machine like the bacterial flagellum, which consists of several distinct parts with specific characteristics, stability is important. During the rotation of the bacterial flagellar motor, which is located in the membrane, the flagella rotate at speeds between 200 and 2000 rpm, depending on the bacterial species. The hook substructure of the bacterial flagellum acts as a universal joint connecting the motor to the flagellar filament. We investigated the formation of the bacterial flagellar hook and its overall stability between the FlgE subunits that make up the hook and attempted to understand how this stability differs between bacteria.

**Results:**

An intrinsically disordered segment plays an important role for overall hook stability and for its structural cohesion during motor rotation. The length of this linker segment depends on the species of bacteria; for *Salmonella enterica* and *Campylobacter jejuni* it is approximately 37 and 54 residues, respectively. Few residues of the linker are conserved and mutating the conserved residues of the linker yields non-flagellated cells. In the case of *Campylobacter*, which rotates its flagella at a speed much higher than that of *Salmonella*, shortening the linker leads to a rupture of the hook at its base, decreasing cell motility. Our experiments show that this segment is required for polymerization and stability of the hook, demonstrating a surprising role for a disordered region in one of the most finely tuned and closely studied macromolecular machines.

**Conclusions:**

This study reveals a detailed functional characteristic of an intrinsically disordered segment in the hook protein. This segment evolved to fulfill a specific role in the formation of the hook, and it is at the core of the stability and flexibility of the hook. Its length is important in the case of bacteria with high-speed rotating flagella. Finding a way of disrupting this linker in *Campylobacter* might help in preventing infections.

**Electronic supplementary material:**

The online version of this article (doi:10.1186/s12915-017-0438-7) contains supplementary material, which is available to authorized users.

## Background

**Fig. 1:**
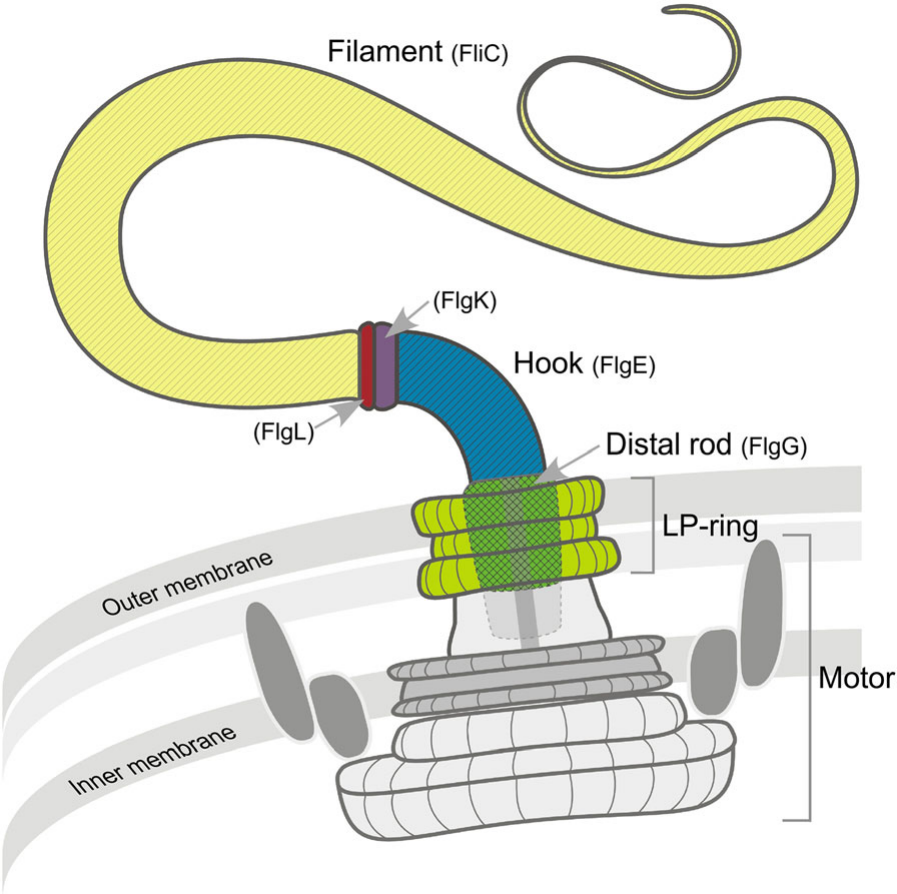
Schematic representation of the flagellum from a gram-negative bacterium. The distal rod (green), located in the outer membrane, is in direct contact with the hook (blue). Between the hook and the filament (yellow) are two junction proteins – FlgK (purple) and FlgL (red). The rod transmits the torque produced by the motor to the hook. During the rotation, the rod is held in place by the LP-ring

**Fig. 2:**
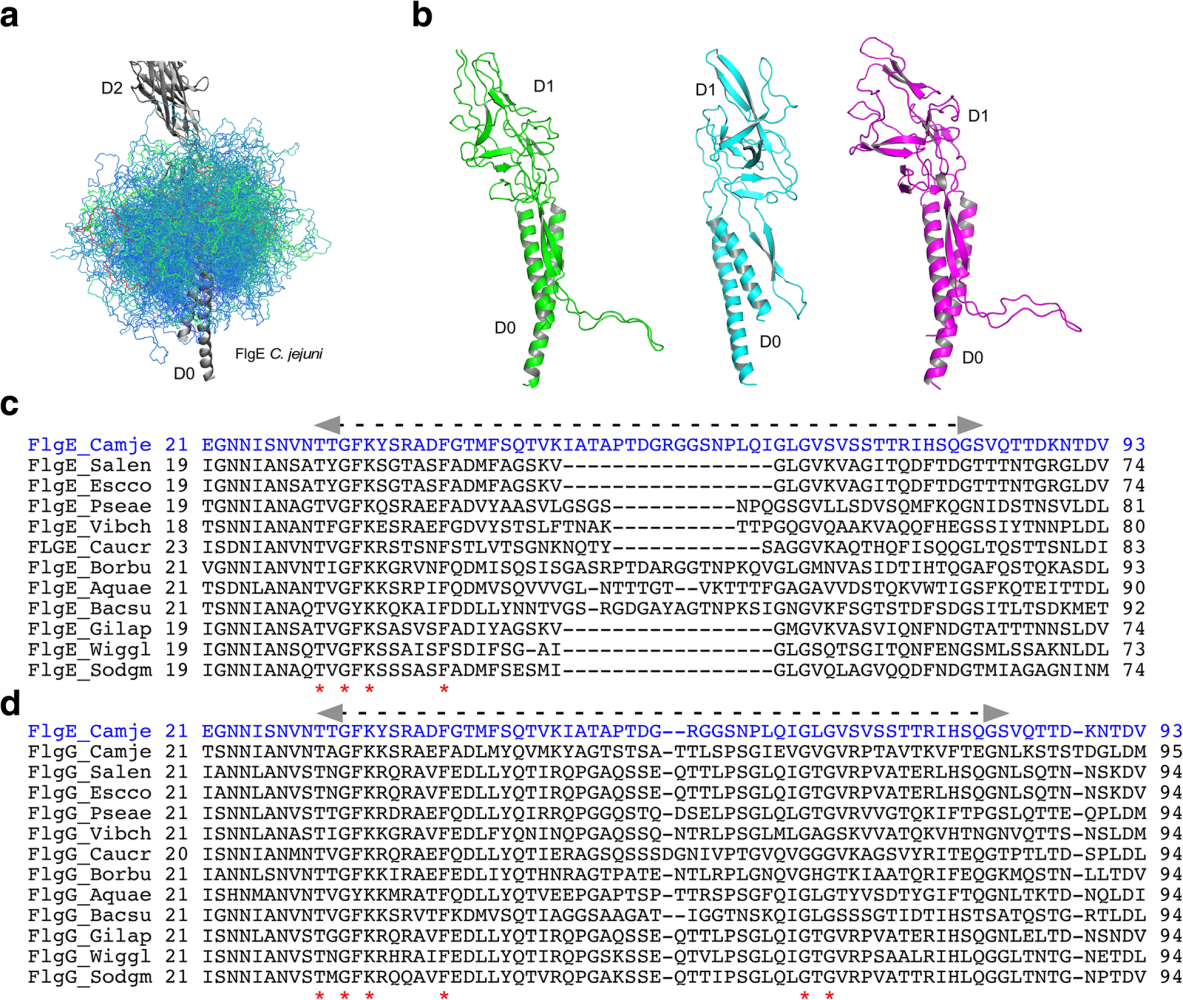
Molecular modeling of the disordered segment and sequence alignment. **a** Molecular modeling calculations showing the flexibility of the disordered segment of FlgE from *C. jejuni*. Representative structures, plotted every 10th structure, are sorted and colored by energy for the loop model population (scale ranges from blue (500 Rosetta energy units; REU) to red (850 REU)). **b** Cartoon representation of the 3D structure of FlgE from *C. jejuni*, in green, with the “L-shaped” (L-Stretch) disordered segment connecting D0 to D1 obtained, and FlgE and FlgG from *S. enterica*, in cyan and in purple, respectively. **c** and **d** Sequence alignments of the rod/hook disordered segment from FlgE and FlgG of gram-negative and -positive bacteria with the position of the disordered segment marked by an arrow. In FlgE (**c**), the segments have different lengths, while in FlgG (**d**), the length is conserved. The red stars show the position of the conserved residues. For comparison, the sequence of FlgE from *C. jejuni* is marked in blue. Camje: *C. jejuni*, Salen: *S. enterica*, Escco: *Escherichia coli*, Pseae: *Pseudomonas aeruginosa*, Vibch: *Vibrio cholerae*, Caucr: *Caulobacter crescentus*, Borbu: *Borrelia burgdorferi*, Aquae: *Aquifex aeolicus*, Bacsu: *Bacillus subtilis*, Gilap: *Gilliamella apicola*, Wiggl: *Wigglesworthia glossinidia*, Sodgm; *Sodalis glossinidius*. Sequence alignment was done with *Clustal Omega* [30]

A protein’s function is linked to its three-dimensional structure [1, 2], which is correlated with its amino acid sequence [3]. However, intrinsically disordered polypeptides have little or no well-defined secondary or tertiary structures, and their functions depend on this lack of structure [4]. In the bacterial flagellum, although ordered domains are important for its function [5–7], a disordered region is found to be crucial for its stability. In flagellated bacteria, the axial part of the flagellum can be divided in three sections made by the filament, the hook, and the rod [8] (Fig. 1). The filament functions like a propeller, the hook is a universal joint that transmits the torque to the filament [8], and the rod is directly connected to the motor located in the membrane and works like a drive-shaft, connecting the motor to the hook [9–11]. While in most bacteria the filament and the hook are made by the assembly of multiple copies of FliC and FlgE, respectively [12, 13], the rod is made by the association of many different proteins [14, 15]. The distal part of the rod is formed by approximately 50 FlgG subunits and is directly in contact with the hook [16]. The structures of FlgE and FlgG proteins showed that two domains, D0 and D1, are structurally conserved [6, 16–19]. In *Campylobacter jejuni*, these domains are connected by a flexible segment previously named the L-stretch because of its shape [19] (Fig. 2a, b, Additional files 1 and 2). This flexible segment that links domains D0 and D1 could not be resolved in FlgE or FlgG structures from *Salmonella enterica* serovar Typhimurium due to experimental limitations [6, 16, 17, 20].

**Fig. 3:**
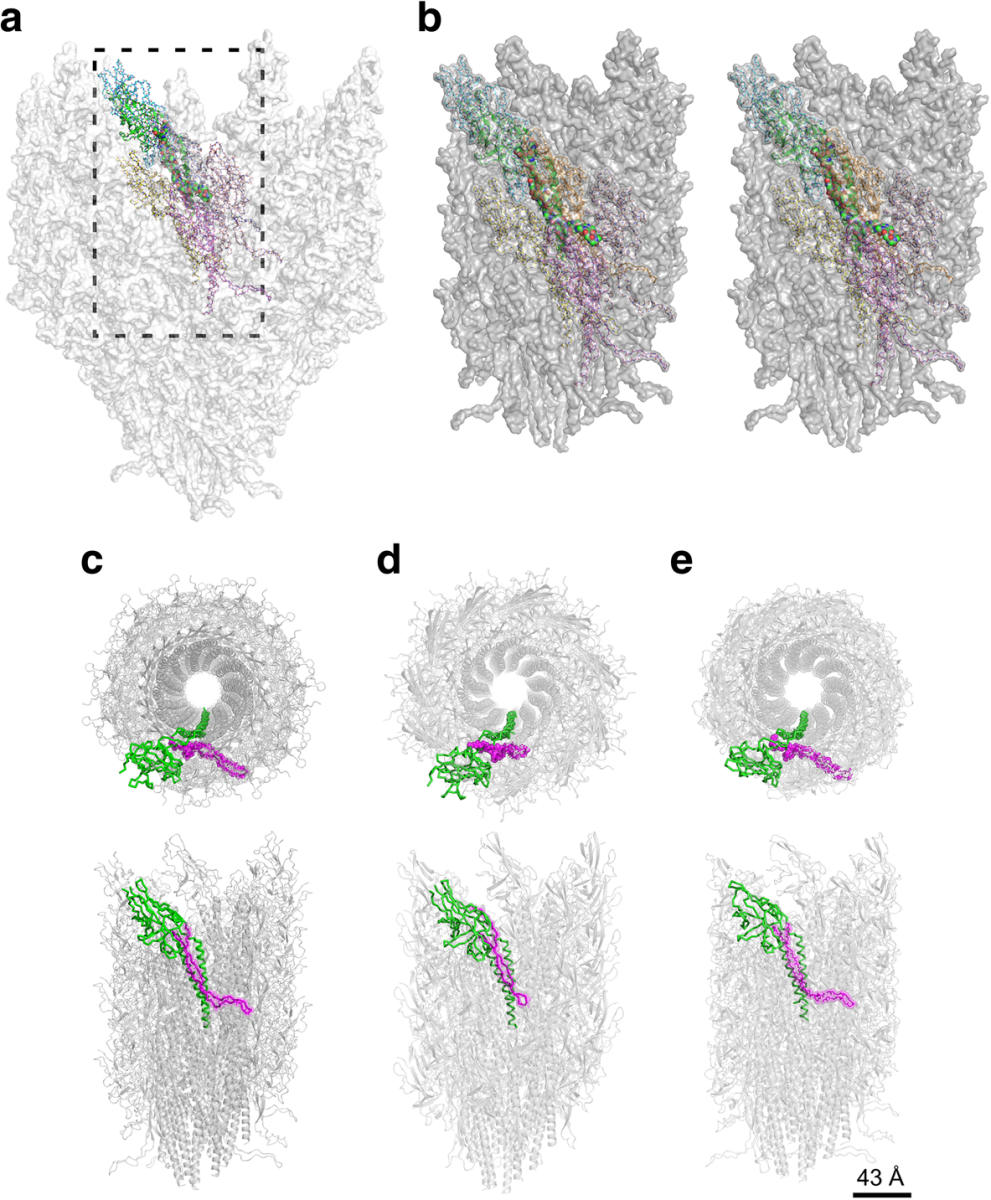
View of the disordered segment in the hook and in the rod. **a** Overview of the hook from *C. jejuni* in grey with six highlighted molecules of FlgE. **b** Zoom-up, stereo view of the core domain of the hook with the disordered segment, represented with spheres, surrounded by molecules of FlgE which it interacts with. Comparative view of domains D0 and D1 of the hook of *C. jejuni* (**c**), namely the hook and the distal rod of *S. enterica* in (**d**) and (**e**), respectively. The molecules that make the hook and the rod are colored in grey with one molecule colored in green and its disordered segment colored in purple. The upper panels in (**c**), (**d**) and (**e**) show a projection from the distal end. In each case, one molecule is colored in green with the ID-Rod-Stretch represented in magenta. Figure prepared with PyMOL (The PyMOL Molecular Graphic System, Schrödinger, LLC. https://www.schrodinger.com/suites/pymol)



Sequence alignments of FlgE and FlgG proteins (Fig. 2c, d) from different bacteria show that the segment linking domains D0 and D1 is conserved in both FlgE and FlgG proteins. Both software prediction of disordered protein segments and molecular modeling calculations indicate that this segment is intrinsically disordered and highly flexible (Fig. 2, Additional file 3: Video S1). Compared to the segment connecting domains D0 and D1 of the bacterial filament protein FliC (Leu31-Asp43), the segment in FlgE does not show any clearly defined native state. For FliC, there is a clearly defined low-energy subpopulation of conformations, corresponding to a well-structured native state, which is absent for FlgE. Three-dimensional structure prediction of this segment in FlgE and FlgG of *S. enterica*, performed with Swiss-Model [21, 22], suggests that it has a disordered structure similar to that of *C. jejuni* FlgE (Fig. 2, Additional file 2). This segment is hereafter named ID-Rod-Stretch for ‘Intrinsically Disordered Rod Stretch’ because it is predominantly found in the rod where it has a conserved length. The ID-Rod-Stretch plays an important role in the formation and stability of the hook. This linker becomes partly structured upon formation of the hook, where it is located in a pocket surrounded by molecules of FlgE (Fig. 3a, b). The ID-Rod-Stretch in the distal rod protein FlgG is similar both in length and structure to that in the hook of *C. jejuni* (Fig. 3c–e), and thus likely shows similar structural behavior. To pinpoint which parts of the ID-Rod-Stretch are important for the assembly and function of the hook, we performed targeted mutagenesis of conserved residues in the disordered segment of FlgE from *S. enterica*. These experiments revealed that this segment is at the center of the stability or flexibility of the hook.

**Additional file 3: Video S1.** Results of the molecular modeling calculations showing the flexibility of the ID-Rod-Stretch of FlgE from *C. jejuni*. (MOV 895 kb)


## Results

### Lys32 of *Salmonella* FlgE is required for hook assembly

**Fig. 4:**
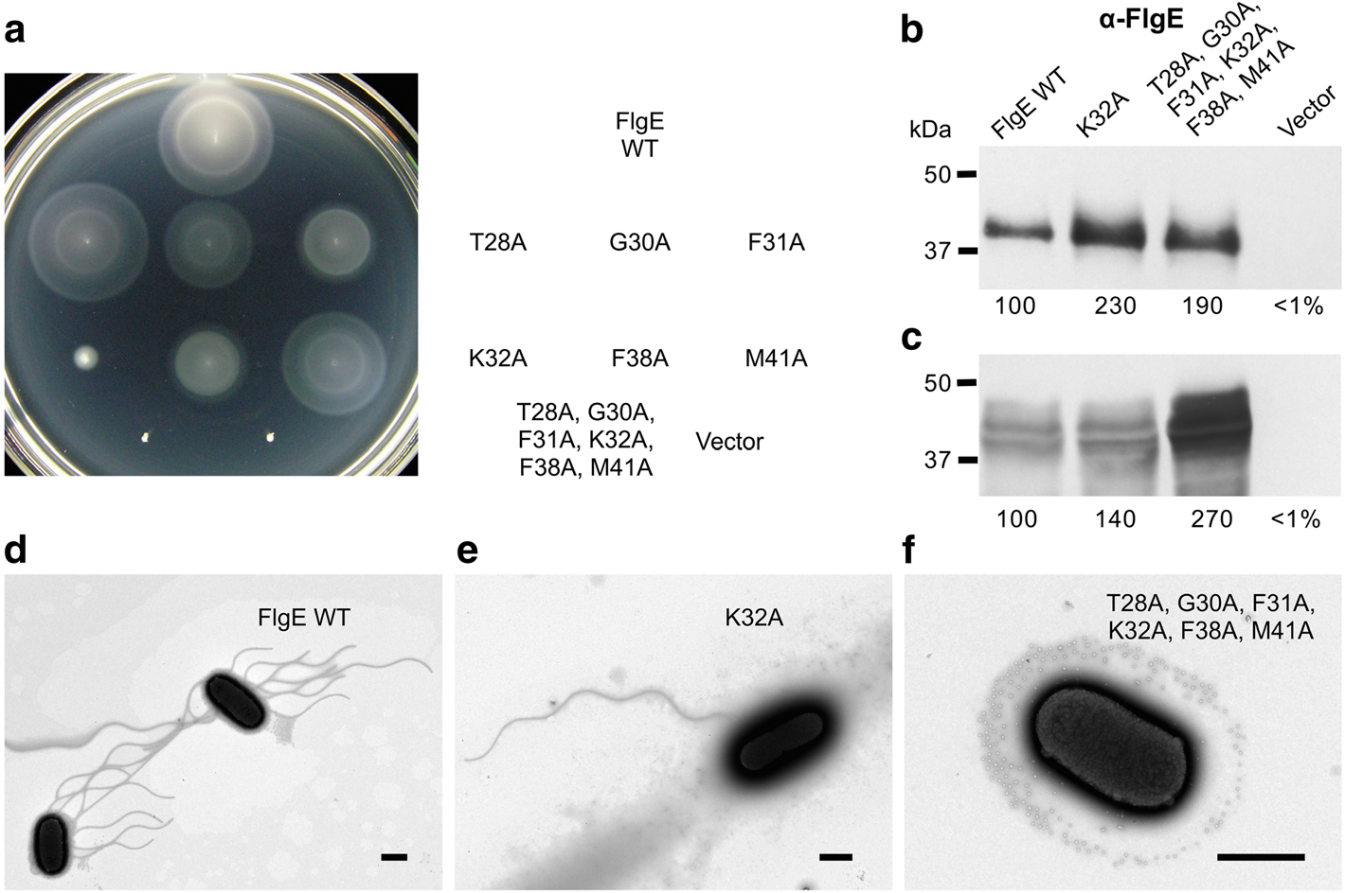
Motility and flagella of *Salmonella* expressing FlgE ID-Rod-Stretch mutant proteins. **a** Motility for strains inoculated into soft tryptone agar that expressed FlgE proteins with conserved amino acid residues exchanged with alanine residues, as indicated. The plate was incubated at 30 °C for 6 h. Western blot analysis of FlgE proteins exported into the supernatant broth (**b**) or remaining in the cell pellet fractions after centrifugation (**c**) for strains grown in LB at 37 °C. Relative band densities (%) compared to wild-type are indicated. Polyclonal antibodies were used. Wild-type FlgE is 42 kDa. Electron micrographs of cells expressing wild-type FlgE (**d**), FlgE K32A (**e**), or FlgE T28A, G30A, F31A, K32A, F38A, and M41A mutant proteins (**f**). Scale bars, 1 μm

In FlgE, the length of the ID-Rod-Stretch is species dependent, while it is conserved in FlgG with a length similar to that of the ID-Rod-Stretch of FlgE from *C. jejuni* with 54 amino acid residues. In FlgE from *S. enterica*, the ID-Rod-Stretch is 37 residues long and goes from Thr28 to Thr64 (Fig. 2c, Additional file 2). Four residues are fully conserved and two are semi-conserved in the N-terminal region of the ID-Rod-Stretch (Fig. 2c, d). In FlgE of *S. enterica*, the four conserved residues correspond to Thr28, Gly30, Lys32 and Phe38, and the two semi-conserved residues correspond to Phe31 and Met41. These six residues were exchanged, individually and all together, with alanine residues in order to examine their importance for the structural integrity and function of the hook. To test the expression of FlgE, a Δ*flgE* null mutant strain was transformed with a plasmid carrying the wild-type *flgE* gene. Motility and flagellar biosynthesis of the transformed cells were similar to wild-type (Additional file 4). The Δ*flgE* mutant strain was transformed with the plasmids carrying the *flgE* genes mutated at codons encoding conserved and semi-conserved residues in the ID-Rod-Stretch and motility of the cells was examined in soft tryptone agar (Fig. 4a). The motility of cells expressing FlgE-K32A was severely decreased compared to cells expressing wild-type FlgE and the other five FlgE proteins with point mutations. The strain, which contained the plasmid encoding FlgE-K32A, expressed and exported excess FlgE, at about twice the amount of wild-type (Fig. 4b, c). The strain containing the plasmid encoding wild-type FlgE produced 12 ± 3 flagella per cell (Fig. 4d). Less than 10% of cells of the strain expressing FlgE-K32A produced flagella, and only one or two flagella per cell were made (Fig. 4e). This suggests that FlgE-K32A protein was mainly being exported un-polymerized.

The strain expressing FlgE with all four conserved and two semi-conserved residues substituted with alanine residues was non-motile due to the absence of flagella (Fig. 4a, f). However, this FlgE mutant protein was synthesized and secreted at more than twice the amount of wild-type FlgE (Fig. 4b, c). This suggests that the FlgE protein with the substitutions T28A, G30A, F31A, K32A, F38A, and M41A was also being exported un-polymerized, which produced a similar but more severe phenotype to that shown with the K32A substitution alone.

**Fig. 5:**
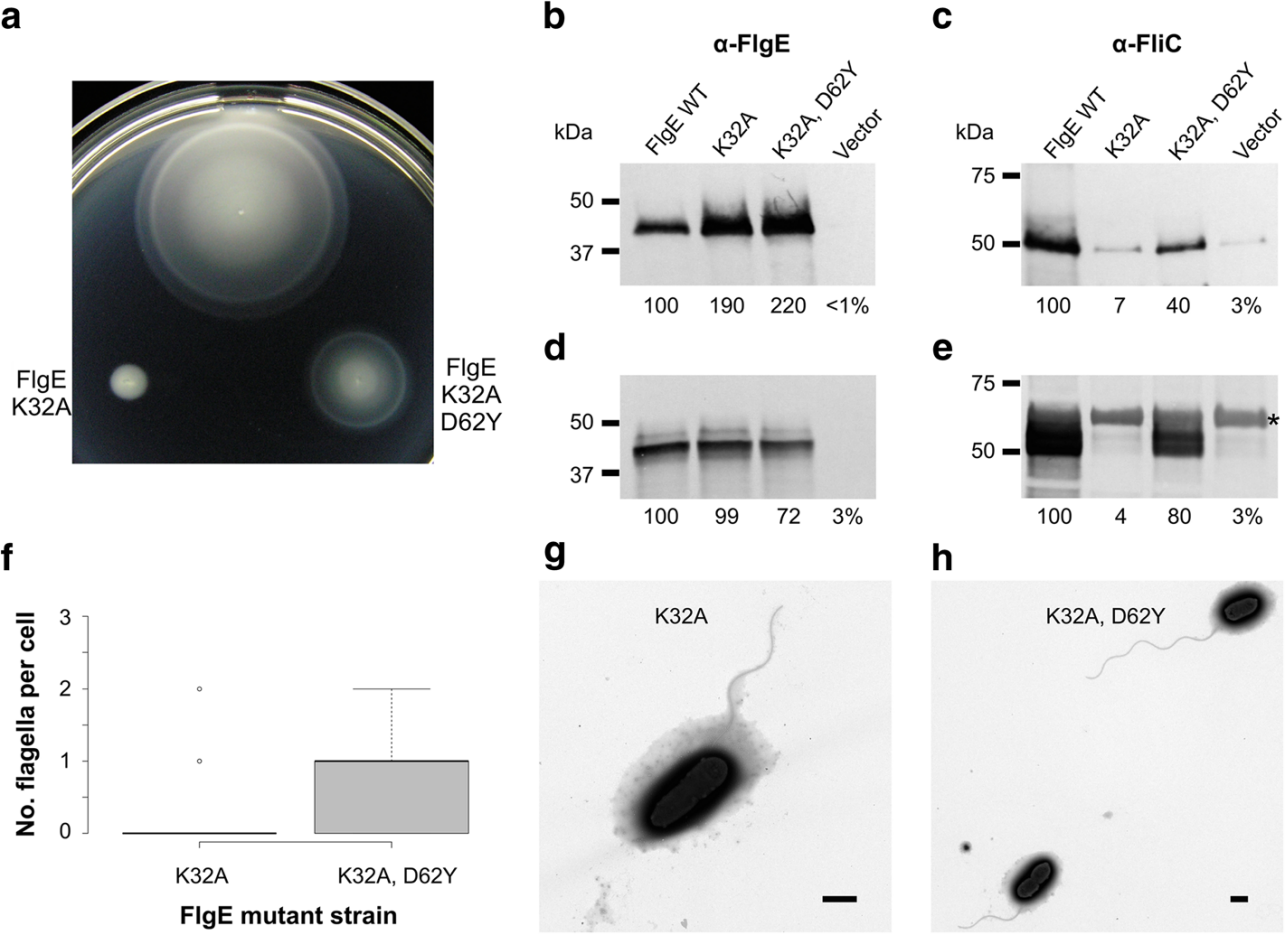
Lys32 of *Salmonella* FlgE is important for hook assembly. **a** Motility in soft tryptone agar of an *S. enterica* Δ*flgE* null mutant strain that harbored plasmids carrying genes encoding the wild-type FlgE protein, a FlgE K32A mutant protein, or a FlgE K32A D62Y double-mutant protein. The plate was incubated for 10 h at 30 °C. Western blot analysis using polyclonal antibodies towards FlgE (**b** and **d**) and FliC (**c** and **e**) proteins exported into the culture supernatant broth (**b** and **c**) or in the cell pellet fractions after centrifugation (**d** and **e**). The bacteria were grown in LB to early stationary phase at 37 °C. Relative band densities (%) compared to FlgE wild-type are indicated. FliC is 51 kDa and wild-type FlgE is 42 kDa. An asterisk in (**e**) indicates a non-specifically bound protein. Box plot (**f**) displaying the distribution of the numbers of flagella per cell, for > 30 cells, and electron micrograph images of cells synthesizing the FlgE K32A mutant protein (**g**) or the FlgE K32A D62Y double-mutant protein (**h**). Bars, 1 μm

Since the FlgE-K32A mutant had decreased functionality, the Δ*flgE* mutant strain harboring the plasmid carrying the *flgE*(*K32A*) mutant gene was incubated in soft tryptone agar for 2–5 days at 30 °C. Three suppressor mutant strains with increased motility were isolated and the colonies were purified. The plasmids, which were extracted and sequenced, showed that the most motile suppressor mutant strain bore an additional mutation in the ID-Rod-Stretch region of the *flgE* gene; it encoded a double-mutant protein FlgE-K32A-D62Y. The motility and flagellar biosynthesis of the Δ*flgE* mutant strain freshly transformed with plasmids carrying *flgE* genes encoding wild-type FlgE, FlgE-K32A, and FlgE-K32A-D62Y was examined (Fig. 5). As expected, motility and export of FliC (flagellin protein) was dramatically reduced in cells expressing FlgE-K32A mutant protein (Fig. 4a–c). With the hook not being made, the feedback loop with the *fliK* gene [23], which informs the cells about the hook completion, keeps sending a signal that the hook has not been completed. The cells, therefore, do not undergo the secretion-specificity switch that normally occurs upon hook completion [23]. Expression of FlgE-K32A-D62Y increased motility and FliC export compared to the FlgE-K32A mutant protein (Fig. 5a, c). However, cells producing the FlgE-K32A-D62Y double-mutant protein made very few flagella, suggesting that it was still mainly being exported un-polymerized (Fig. 5). The obtained data demonstrate that Lys32 is important for assembly of the flagellar hook of *S. enterica*.

### The ID-Rod-Stretch is required for hook assembly and mobility

To study the role of the ID-Rod-Stretch in the assembly and function of the hook, we divided it into short segments of five amino acid residues and mutated them by exchanging them with alanine residues. We assumed that it would reveal the regions that are required for hook structural integrity and those that are required for hook mobility. The first five residues mutated were Ala27-Thr28-Tyr29-Gly30-Phe31, the second set of five residues mutated were Lys32-Ser33-Gly34-Thr35-Ala36, and so on. In some cases, the residue was already alanine. The results are summarized in Additional file 5.

**Fig. 6:**
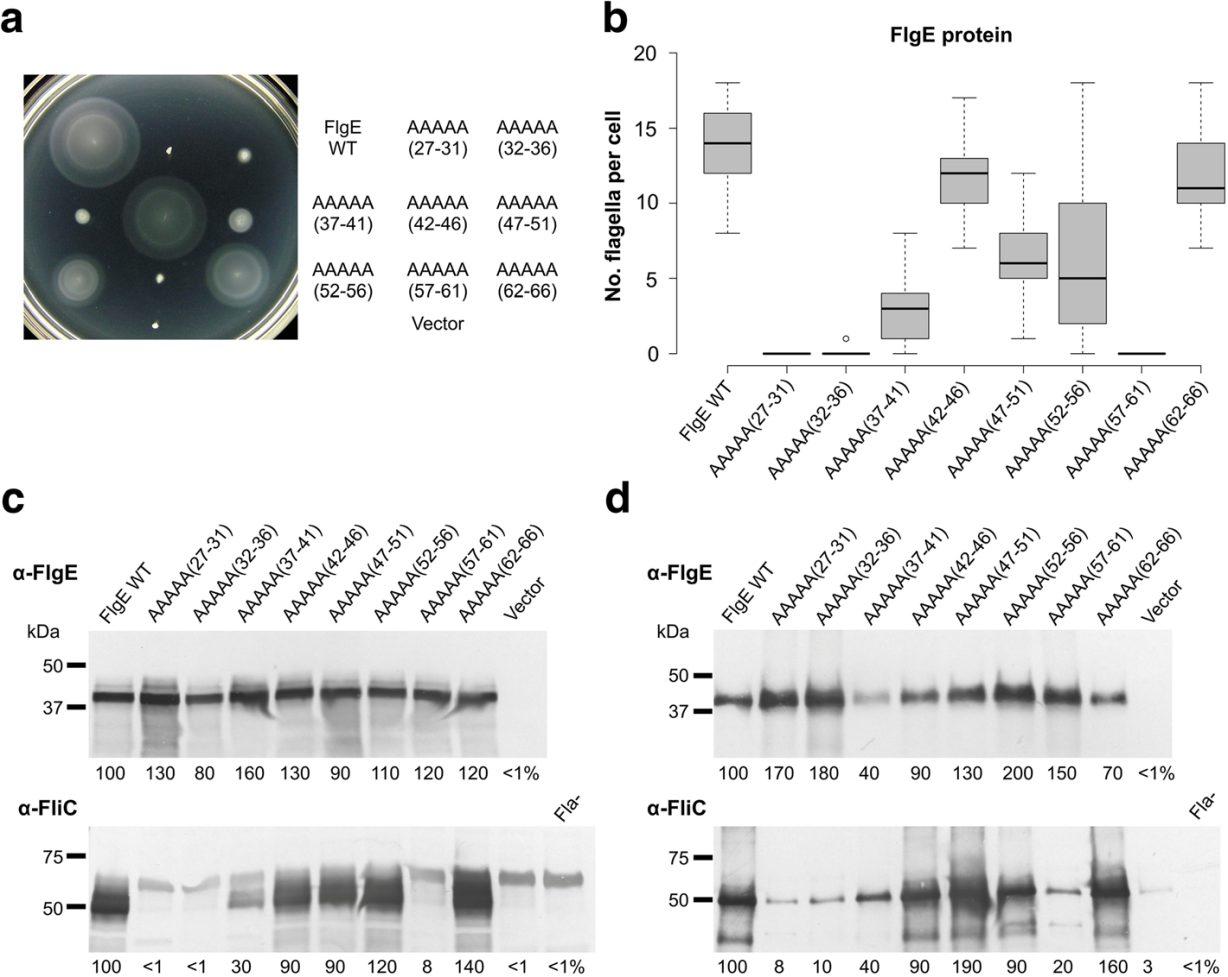
Motility and biosynthesis of flagella is decreased for *Salmonella* ID-Rod-Stretch mutant strains. **a** Motility phenotype in soft tryptone agar of a Δ*flgE* null mutant strain that harbored plasmids carrying *flgE* mutant genes. FlgE proteins were encoded with five amino acid residues exchanged with five alanine residues. The plate was incubated at 30 °C for 6 h. Box plot (**b**) displaying the distribution of the numbers of flagella per cell, for > 30 cells of each strain examined using transmission electron microscopy. Western blotting analysis of the amount of FlgE and FliC in the cell (**c**) and exported (**d**) into the culture supernatant broth for cultures of the strains grown in LB to early stationary phase at 37 °C. Polyclonal antibodies were used. The Fla- strain does not produce flagella. Relative band densities (%) compared to wild-type are indicated. FliC is 51 kDa and wild-type FlgE is 42 kDa

Strains that expressed FlgE AAAAA(27–31) and FlgE AAAAA(32–36) mutant proteins were non-motile, exported excess FlgE, synthesized little FliC, and did not produce flagella (Fig. 6). For the strain that expressed the FlgE AAAAA(32–36) mutant protein, a single flagellum was made by < 5% of cells. This region is unstructured and at the N-terminus of the ID-Rod-Stretch. The strain that synthesized the FlgE AAAAA(57–61) mutant protein was non-motile, exported excess FlgE, made very little FliC, and did not produce flagella (Fig. 6). This region is unstructured and at the C-terminus of the ID-Rod-Stretch. However, the FlgE AAAAA(62–66) mutant protein was able to function almost as well as wild-type FlgE, which demonstrates that the amino acid composition at the very C-terminal region of the ID-Rod-Stretch is not critical.

The strains that synthesized the FlgE AAAAA(42–46) mutant protein was fully motile and made flagella normally (Fig. 6a, b). These amino acid residues are located in the middle of the ID-Rod-Stretch in the connecting loop region (Additional file 2). Strains that synthesized FlgE AAAAA(37–41), FlgE AAAAA(47–51), and FlgE AAAAA(52–6) mutant proteins were significantly less motile than wild-type and produced decreased amounts of flagella (Fig. 6, Additional file 6). These mutants include the residues that comprise the two ID-Rod-Stretch N- and C-terminal region anti-parallel β-strands. Examination by dark-field microscopy revealed that some cells within the populations were motile, but were less so than wild-type. The amount of the FlgE AAAAA(37–41) mutant protein in the supernatant broth was approximately 40% that of wild-type (Fig. 6d) and yet the amount of this protein in the cell pellet was slightly more than in wild-type (Fig. 6c). If cells do not undergo a switch from rod/hook-type export substrates to filament-type export substrates [23], FlgE will continue to be produced, which may account for more FlgE than wild-type.

### *Campylobacter* FlgE has a long ID-Rod-Stretch vital for hook stability

The ID-Rod-Stretch of FlgE from *C. jejuni* strain 81116 has an insertion of approximately 20 residues in its middle compared to *S. enterica* LT2 FlgE. This region was shown to be involved in inter-subunit interactions in the hook [19]. To study the importance of this insertion, a strain of *C. jejuni* was made where the *flgE* gene was changed by deleting the codons for 21 amino acid residues from Gln46 to Ile66. The deletion was designed based on the structure of *C. jejuni* FlgE [19].

**Fig. 7:**
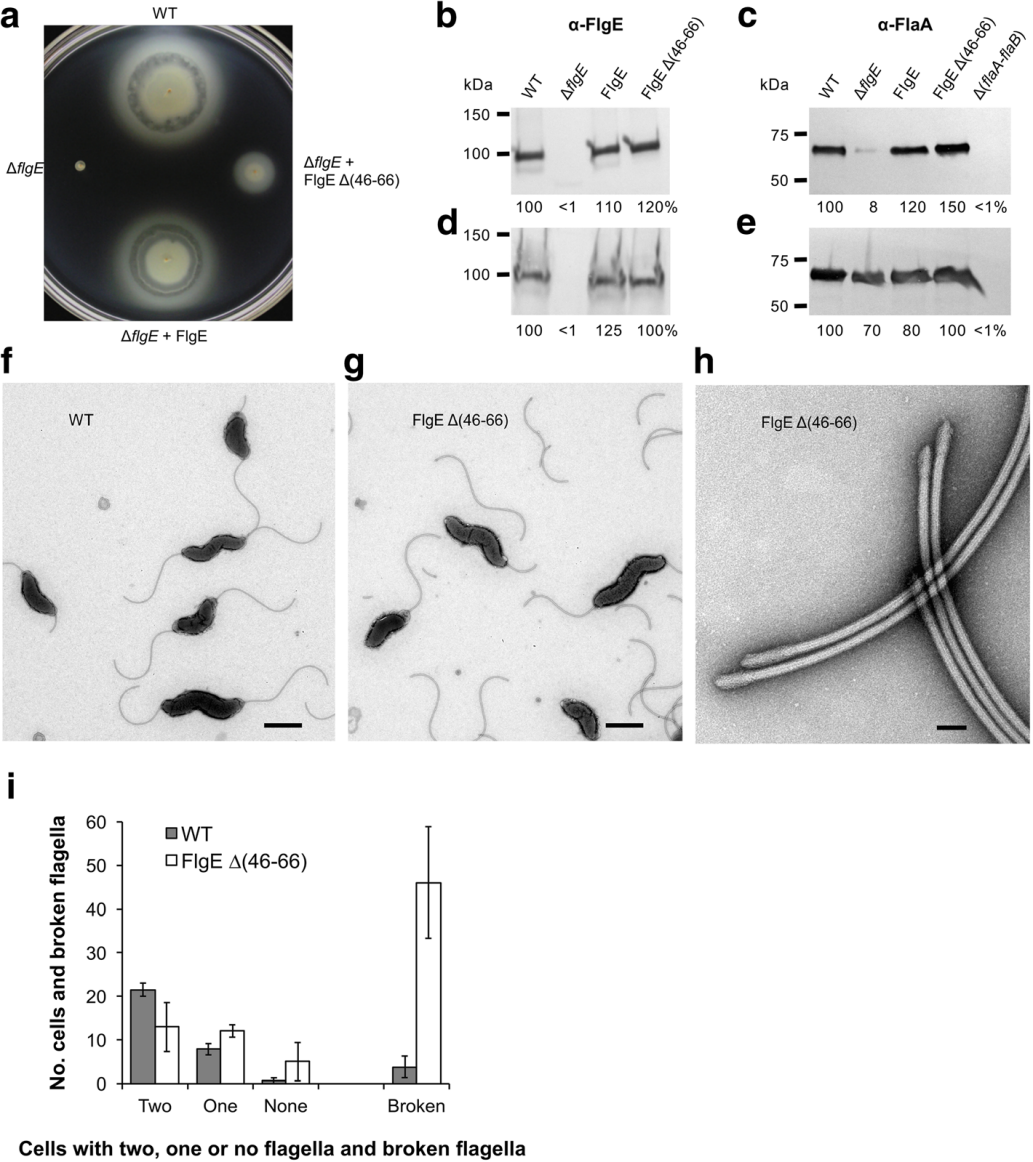
Decreased motility and flagellar breakage for a *Campylobacter* ID-Rod-Stretch deletion mutant strain. **a** Motility for a wild-type strain, a Δ*flgE* mutant strain, a Δ*flgE* mutant strain encoding an inserted copy of the wild-type *flgE* gene, and a Δ*flgE* mutant strain encoding a gene for a FlgE mutant protein deleted for amino acid residues 46–66, inoculated into Mueller–Hinton motility media. The plate was incubated at 42 °C for 24 h. Western blotting analysis of FlgE (**b**) and FlaA (flagellin) (**c**) proteins exported into the culture supernatant, and FlgE (**d**) and FlaA (**e**) protein synthesis for whole cells, using polyclonal antibodies. Cultures of the strains were grown in Mueller–Hinton broth at 42 °C to stationary phase. Relative band densities (%) compared to wild-type are indicated. Electron micrographs at 2000× magnification of *C. jejuni* expressing wild-type FlgE (**f**) and FlgE Δ(46–66) (**g**). Scale bars, 1 μm. **h** Electron micrograph at 25,000× magnification of flagella broken at the hook for cells expressing FlgE Δ(46–66). Scale bar, 50 nm. **i** Quantification of flagella. Cells with and without flagella and surrounding broken flagella were counted at 1000× magnification for at least 30 cells on three carbon grids for the wild-type and FlgE Δ(46–66) strains. The numbers were normalized to 30 cells per grid and mean average numbers per grid and standard deviations are shown

Motility in Mueller–Hinton motility media at 42 °C and the flagellar assembly phenotypes of four strains of *C. jejuni* were examined (Fig. 7a–e). The four strains were the wild-type strain, a Δ*flgE* (Δ*flgE*::Km^R^) deletion mutant strain, a Δ*flgE* + FlgE pseudo-wild-type strain, and a Δ*flgE* + FlgE Δ(46-66) ID-Rod-Stretch mutant strain. The Δ*flgE* deletion mutant strain was non-motile and did not produce flagella. The small amount of flagellin (8%) released by the Δ*flgE* mutant strain was presumably due to cell lysis (Fig. 7c). The pseudo-wild-type strain, which bore a Δ*flgE* gene deletion and encoded the wild-type *flgE* gene integrated into the rRNA gene cluster, was fully motile and assembled flagella normally. The Δ*flgE* + FlgE Δ(46–66) ID-Rod-Stretch mutant strain was much less motile than the wild-type strain (Fig. 7a), yet it synthesized and exported FlgE and flagellin at similar amounts to the wild-type strain (Fig. 7b–e). However, when negatively stained cells of the Δ*flgE* + FlgE Δ(46–66) ID-Rod-Stretch mutant strain were examined by transmission electron microscopy, many broken flagella were seen on the grid surface compared to the wild-type strain at 2000× magnification (Fig. 7f, g). At 25,000× magnification, it was revealed that the flagella of the Δ*flgE* + FlgE Δ(46–66) ID-Rod-Stretch mutant strain were breaking at the hook (Fig. 7 h). The surfaces of several electron microscope grids were scanned at low power (1000×) magnification for each strain. A surface area covering at least 30 evenly distributed cells was scanned for each grid. The number of cells with one, two, or no flagella and free broken flagella were counted for each grid and the numbers were normalized to 30 cells for each grid. Compared to wild-type strain, there were more than ten times the number of broken flagella with the FlgE Δ(46–66) ID-Rod-Stretch deletion mutant strain (Fig. 7i).

Based on the micrographs that we have of the wild-type unbroken hook attached to cells and the broken hooks of the ID-Rod-Stretch deletion mutant strain, the wild-type hook of *C. jejuni* has a mean length of 105 ± 8 nm (*N* = 7), while the broken hooks have an average length of 85 ± 15 nm (*N* = 23). Almost intact hooks were attached to the filament. We did not see any breakage of the hook without filament, which makes us suspect that, in the absence of the filament, the hook will probably not break. The point of breakage is close to the rod-hook junction. During the growth of the flagellum, the hook assembles normally, followed by the completion of the flagellum. In the case of a shortened ID-Rod-Stretch in the hook, interactions between FlgE and FlgG proteins at the rod-hook interface and between FlgE proteins in the hook are reduced. As the filament is being assembled, the load on the hook gradually weakens the rod-hook connection, inducing breakage of the hook.

## Discussion

Intrinsically disordered peptides often interact with many different proteins [24] and are known for their involvement in important processes in cells [25]. Mutations in the disordered regions induce a reduction in these interactions as well as dysfunction in cells, which often lead to diseases. In the bacterial flagellum, the ID-Rod-Stretch will not only connect two different domains within the protein, but will also interact with several neighboring molecules in both the hook and rod.

**Fig. 8:**
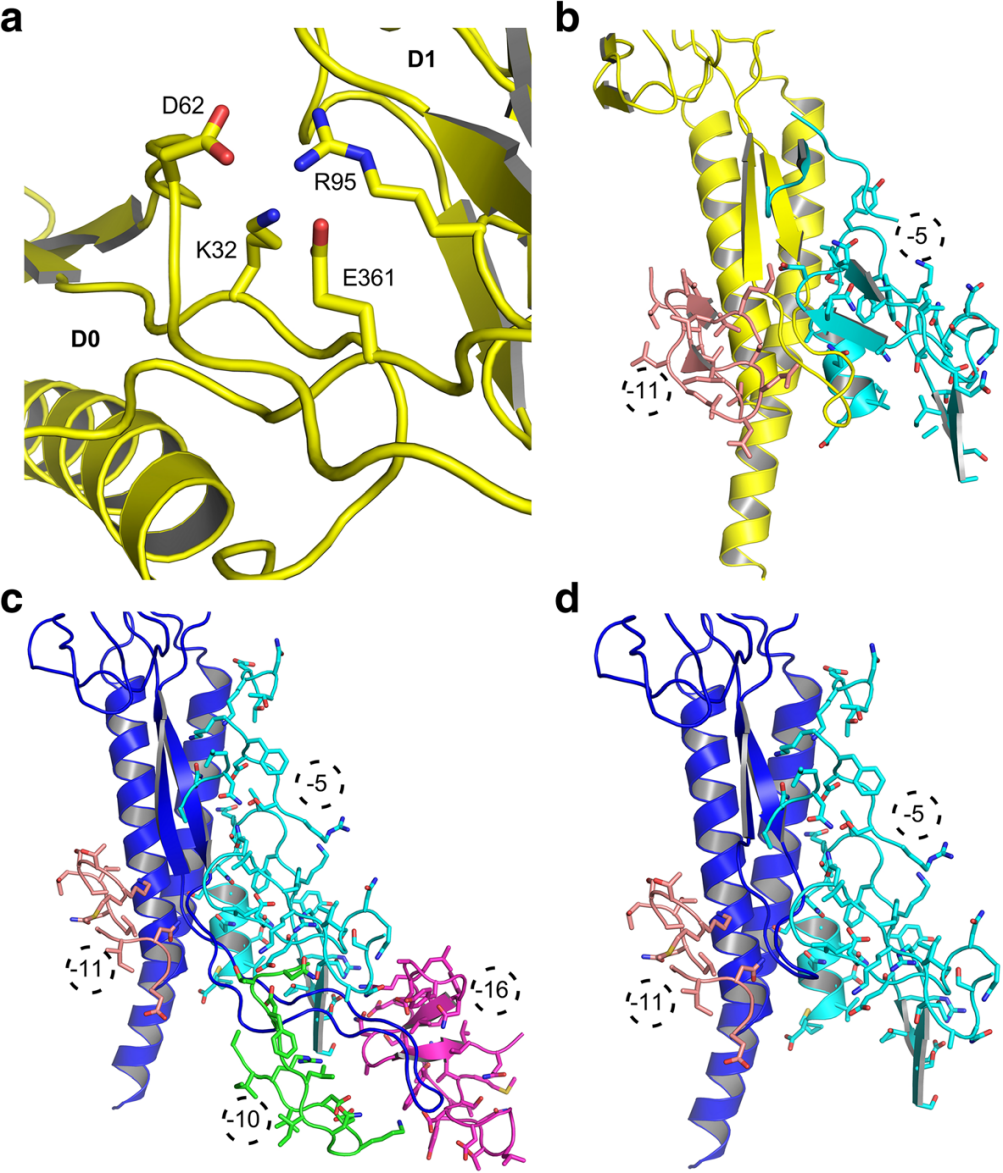
Analysis of the effect of mutations in the structural context of the hook. Analysis of the different mutations in the ID-Rod-Stretch: (**a**) Lys32 of *S. enterica* FlgE, a conserved residue of the ID-Rod-Stretch, is located in a pocket with Asp62, Arg95, and Glu361 in this theoretical model. Both Arg95 and Glu361 are conserved in the FlgE protein family. **b** Two of the three molecules of FlgE, at position “-5” (cyan) and “-11” (light brown), which interact with the modeled ID-Rod-Stretch of the flagellar hook of *S. enterica*. Mutations in the ID-Rod-Stretch that are in the interactive domains will affect the stability of the hook. The cryo-electron microscopy structure, including ID-Rod-Stretch of FlgE from *C. jejuni* [19] (**c**), is longer than in *S. enterica* and it is shown here interacting with four FlgE molecules in positions “-5” (cyan), “-10” (green), “-11” (light brown), and “-16” (purple). **d** Reducing the length of the disordered segment of *C. jejuni* FlgE reduces its number of interactions with other FlgE molecules in the hook. This has a negative effect on the cohesion of the hook

Compared to *C. jejuni*, the ID-Rod-Stretch of FlgE from *S. enterica* undergoes fewer interactions with other molecules of FlgE in the hook because of its shorter amino acid length (Figs. 2 and 8). Among mutations of the conserved residues, the mutations T28A and M41A do not have any effect on the growth of the flagellum and on bacterial motility. The substitution of the conserved Lys32 by an alanine in FlgE from *S. enterica* drastically decreases flagellar biosynthesis. Based on the structure of FlgE from *C. jejuni* and on the model of the ID-Rod-Stretch of *S. enterica* obtained by homology modeling using Swiss-Model [21, 22], Lys32 in FlgE from *S. enterica* has contacts with Asp62, in the ID-Rod-Stretch, and with Arg95 and Glu361 both located in domain D1 of the same molecule (Fig. 8a, Additional file 7). Similar contacts are found in the structure of FlgE from *C. jejuni* [19], with residue Lys34 in close contact with Gln81 and Arg115 and forming a salt bridge with Glu809; these four residues respectively correspond to Lys32, Asp62, Arg95, and Glu361 of FlgE from *S. enterica*. Residues Lys32, Arg95, and Glu361 are highly conserved in bacterial FlgE proteins. The K32A mutation seems to disrupt the interactive pocket of Lys32 that coordinates the connection between the N-terminal region of the ID-Rod-Stretch with domain D1 (Fig. 8a).

Both mutations F31A and F38A decrease the motility, but less severely than the effect of the mutation K32A. However, the exchange of all four conserved amino acid residues, Thr28, Gly30, Lys32, and Phe38, and the two semi-conserved residues, Phe31 and Met41, of the ID-Rod-Stretch with alanine residues has a cumulative effect that results in non-flagellated cells of *S. enterica*. Among these residues, Thr28, Gly30 and Phe31 are close to Lys32. Substitutions to alanine residues at these positions accentuate the effect of the K32A substitution, leading to non-flagellated cells. These mutations should have the same effect on all ID-Rod-Stretch with lengths similar to that of *S. enterica*.

The ID-Rod-Stretch can be divided in two classes. The first class, made of short ID-Rod-Stretch, is found only within some FlgE proteins, such as in *S. enterica*, and is made of approximately 30 amino acid residues (Additional file 2). The second class, with long ID-Rod-Stretch, is found in all FlgG proteins and in a few FlgE proteins, such as in *C. jejuni*, and is made of approximately 60 amino acid residues (Additional file 2). Mutating the conserved residues from the short ID-Rod-Stretch will produce non-flagellated, non-motile cells. However, these mutations will not have the same drastic effect on the long ID-Rod-Stretch due to the extensive set of interactions with other molecules (Fig. 8c). These interactions do not exist in the case of a short ID-Rod-Stretch.

*C. jejuni* strains encoding FlgE AAAAA(27–31) and FlgE AAAAA(32–36) mutant proteins are non-motile for similar reasons. These amino acid substitutions affect some of the conserved residues and might directly affect the stability of the β-strands. These regions of the ID-Rod-Stretch are very important for the assembly of the hook. The strain encoding FlgE AAAAA(42–46) is fully motile because, in *S. enterica*, amino acid residues Phe42 to Lys46 of the ID-Rod-Stretch do not interact with other FlgE molecules. We predict that this will be the same for all bacteria with a ID-Rod-Stretch of similar length to that of *S. enterica*. The strains that produce FlgE with the mutations FlgE AAAAA(37–41), FlgE AAAAA(47–51), and FlgE AAAAA(52–56) are significantly less motile than wild-type because these segments of the ID-Rod-Stretch play important roles in the interaction with other subunits of FlgE in the hook (Fig. 8b).

In the hook of *S. enterica*, the ID-Rod-Stretch of FlgE interacts with three molecules of FlgE, while in the hook of *C. jejuni*, the ID-Rod-Stretch of FlgE interacts with five molecules of FlgE (Fig. 8b, c, Additional file 7). In FlgE from *C. jejuni*, the ID-Rod-Stretch is 20 residues longer than that of *S. enterica*. Deletion of these extra residues prevents interactions with two molecules of FlgE from different protofilaments (Fig. 8d). One of these molecules is located in the neighboring protofilament at the “-16” position, while the second molecule is located two protofilaments away at the “-10” position (Fig. 8c, d). These interactions cannot be found in the hook of bacteria with a shorter ID-Rod-Stretch, such as *S. enterica* (Fig. 8b), since their ID-Rod-Stretch does not extend to interact with more FlgE molecules due to a shorter amino acid sequence. Disrupting these sets of interactions reduces the cohesion of the hook of *C. jejuni*. In *C. jejuni* cells expressing FlgE with a shorter ID-Rod-Stretch, flagella, while being assembled normally, detach themselves from the cell by breaking at the site of the hook (Fig. 7). Bacterial flagellar motors rotate at between 100 and 2000 Hz [26], and the hook undergoes conformational changes while rotating about its axis [27, 28]. The interactions between FlgE molecules must ensure the stability of the hook while enabling these conformational changes. The intrinsic disorder of the long *C. jejuni* ID-Rod-Stretch may be particularly important in this context for enabling adjacent hook monomers to maintain favorable interactions throughout the variety of positions and of conformations encountered during rotation, by providing multiple, nearly isoenergetic conformations that can be accessed as the hook rotates. The flagellar motor of *C. jejuni* is known to produce a very high torque compared to the flagellar motor of other bacteria [29]. This high torque could be the reason behind FlgE having a long ID-Rod-Stretch. In a recent study, Fujii et al. [16] increased the length of the ID-Rod-Stretch of the hook of *S. enterica* through the insertion of 18 residues taken from the distal rod protein, FlgG, of *S. enterica*. The authors found that the polyhook assessed became as straight as the rod, concluding that the hook became as rigid as the rod. Our explanation is that the hook became less flexible due to this insertion, but not as rigid as the rod. We have shown that the ID-Rod-Stretch is at the center of stability and flexibility of the hook. However, there is a difference between the reduced flexibility and the rigidity found in the rod. The latter may be, partly, due to the presence of a long ID-Rod-Stretch. In our study, the native hook of *C. jejuni*, with its ID-Rod-Stretch similar to that found in the rod, is fully motile and flexible. If the hook of *Campylobacter* was as rigid as the rod, it would not have been able to function as a universal joint and the flagellum would not have been able to rotate about its axis. The length of the ID-Rod-Stretch does not change the flexibility of the hook such as to induce a rigid structure similar to that of the rod.

More generally, deleting approximately 20 residues that are inserted in the long ID-Rod-Stretch in both FlgE or FlgG will reduce the interactions between molecules that stabilize the hook or the distal rod, as shown here for the case of *C. jejuni* (Figs. 7 and 8c, d). This reduction of interactions will destabilize the hook, or the rod, and the flagellum may be ripped off from its base (Fig. 7 h, i).

## Conclusion

The hook disordered segment is important for both the formation and stability of the hook, and thus for cell motility. In the case of pathogenic bacteria, such as *C. jejuni*, which also uses its flagella to secrete toxins, we believe that targeting the ID-Rod-Stretch could be a strategy to prevent toxin secretion into host cells.

## Methods

### Protein sequence alignment

Sequence alignment was performed with Clustal Omega [30].

### Disorder prediction of the ID-Rod-Stretch

Prediction of disordered regions was performed using online software Predictor of Natural Disordered Regions (PONDR) [31]. The prediction shows that a part of the segment of *C. jejuni* FlgE, residues Thr55 to Arg77, has the highest probability of being intrinsically disordered.

### ID-Rod-Stretch modeling

To assess the level of disorder present in the D0–D1 linker of various flagellar components, we generated and analyzed an ensemble of optimized loop structures using the *loopmodel* module of Rosetta 2016.46.59086 [32]. Fragment libraries were generated for the full-length protein of each target using the Robetta web server [33], and thousands of loop models (2000 for *S. enterica* FlgE and 3500 for *C* *.* *jejuni* FlgE) were subsequently obtained using the quick_ccd modeling method and refine_ccd refinement method. We generated loop models for *S. enterica* FlgE (residues 25–64), *C. jejuni* FlgE (residues 30–82), and *S. enterica* FliC (residues 30–44), each in the context of a single full-length protein monomer. Built models were aligned to the corresponding initial structure by the D0 helices (which had been kept rigid during the modeling process), and Cα RMSDs calculated between the aligned, modeled loop and the initial structure using the MDAnalysis package [34]. Structural figures and movies were generated using VMD 1.9.3 [35].

### Strains and culture conditions

Bacterial strains and plasmids used in this study are listed in Additional file 8. The following strains and plasmids have been previously described: *S. enterica* strains SJW1103 [36], JR501 [37], and SJW1368 [38]; *C. jejuni* strains 81116 [39], CB991 [19], and CB-A9 [19]; and plasmids pKD13 [40], pKD46 [40], pCP20 [41]; pTrc99A-FF4 [42], and pCB956 [19]. *S. enterica* and *E. coli* strains were cultured using “Luria–Bertani” broth (LB) or agar at 37 °C [43]. *C. jejuni* strains were cultured on Mueller–Hinton agar (Difco, Detroit, MI, USA) and incubated at 42 °C under microaerophilic conditions (85% N_2_, 10% CO_2_, and 5% O_2_) in a Tri-Gas incubator. For *E. coli*, ampicillin (50 μg mL^–1^) was added to media where appropriate. For *Salmonella*, kanamycin (50 μg mL^–1^) and ampicillin (100 μg mL^–1^) were added to media as required. For *Campylobacter*, trimethoprim (5 μg mL^–1^), vancomycin (10 μg mL^–1^), kanamycin (50 μg mL^–1^), and apramycin (60 μg mL^–1^) antibiotics were used where appropriate.

### Plasmids

Oligonucleotides used in the plasmid constructions are listed in Additional file 9. The *Salmonella flgE* expression plasmid pCB954 was made as follows: the *flgE* gene was amplified in a PCR reaction with primers Fd-NdeI-flgESe and Rv-BamHI-flgESe and strain SJW1103 genomic DNA template. The PCR product and plasmid pTrc99A-FF4 were digested with Nde I and BamH I restriction enzymes. The digested PCR product and plasmid DNA were ligated using T4 DNA ligase. Other plasmids were derived using site-directed mutagenesis with QuikChange Lightning site-directed mutagenesis kits (Agilent, USA). Standard molecular biology procedures were followed [43].

### Construction of *Salmonella* mutant strains

To make a Δ*flgE*::FRT null mutant strain the Lambda Red homologous recombination method of Datsenko and Wanner [40] was used. Briefly, an FLP-flanked kananamycin-resistance cassette with ends homologous to *flgE* gene was amplified in a PCR reaction with primers Fd-flgESe-FKF and Rv-flgESe-FKF and plasmid pKD13 as template.

### Construction of *Campylobacter* mutant strains

To make the *C. jejuni* FlgE ID-Rod-Stretch deletion-mutant strain CB-A137, the parent strain CB991, bearing a Δ*flgE*::Km^R^ allele, was transformed naturally with pCB-A128 suicide vector DNA, as previously described [19, 44]. PCR was used to confirm that double-crossover homologous recombination had occurred within the rRNA cluster. PCR products were sequenced by chain-termination dideoxynucleotide sequencing using a BigDye Terminator v3.1 cycle sequencing kit (Thermo Fisher Scientific, USA). Strain CB-A137 encodes a *flgE* ID-Rod-Stretch deletion mutant gene within the rRNA gene cluster. The rRNA gene cluster has been previously determined to be an appropriate location for insertion of genes for stable gene expression in *C. jejuni* [45, 46].

### Motility assays

Motility of *Salmonella* strains were examined using soft tryptone agar plates consisting of 0.35% w/v agar containing the appropriate antibiotics [47]. Plates were stab-inoculated with colonies of a fresh transformation and incubated at 30 °C for the desired time. Experiments were repeated at least four times for each strain. For the isolation of suppressor mutant strains with increased motility, colonies were streaked through the soft tryptone agar plate and incubated at 30 °C for up to 5 days. The suppressor mutant strains were purified by inoculation onto fresh media.

Motility of *Campylobacter* was examined in Mueller–Hinton motility media, which contained 0.4% w/v agar [48]. The strains were grown in Mueller–Hinton broth at 42 °C for 24 h, the optical density of each culture was normalized to OD_600_ nm 0.5 and 1 μL was stab-inoculated into Mueller–Hinton motility media. Motility phenotypes were examined after incubation of strains at 42 °C for the desired time.

### Flagellar protein immunoblotting

Immunoblotting of exported flagellar proteins and proteins remaining in the cell pellet was performed similarly as described previously [49, 50]. For *Salmonella*, cultures were grown in LB at 37 °C until early stationary phase (OD_600_ nm 1.5). Culture broth (1.5 mL) was sampled and centrifuged at 10,000× *g* for 10 min. Cell pellets were frozen, and 1.4 mL of supernatant broth was put into a clean tube and centrifuged at 20,400× *g* for 30 min. Clarified supernatant broth (1.3 mL) was taken and 150 μL of trichloroacetic acid solution was added to precipitate proteins. Supernatant solution samples were centrifuged at 16,100× *g* for 30 min in order to obtain protein pellets. The protein pellets from the supernatant solutions and thawed cell pellets were suspended in Tris.Cl-SDS buffer or 2× SDS loading buffer (7 M Urea, 0.1 M Tris.Cl (pH 6.8), 140 mM sodium dodecyl sulfate, 5% v/v *β*-mercaptoethanol, 0.2% w/v bromophenol blue) to an equivalent of 27 μL mL^–1^, at OD_600 nm_ 1.5 (i.e. the cell pellets were suspended in 40 μL 2× SDS loading buffer).

For *Campylobacter*, strains were grown in Mueller–Hinton broth containing antibiotics as required and grown at 42 °C under microaerophilic conditions for 24 h. The optical density varied from culture to culture between OD_600_ nm 0.4 and 1.2. Cells and proteins from the supernatant broth were harvested by centrifugation of 1.5 mL culture broth, as described above for *Salmonella* cultures. The proteins from the supernatant solutions and cell pellets were suspended in Tris.Cl-SDS buffer or 2× SDS loading buffer to an equivalent of 80 μL mL^–1^, at OD_600 nm_ 1.0.

Samples were loaded onto the wells of a 4–20% w/v Mini-PROTEAN TGX polyacrylamide gel (Bio-Rad, USA) and subjected to electrophoresis at 120 V for approximately 1 h. Western Breeze chromogenic detection kits were used to detect proteins following manufacturer’s instructions (Thermo Fisher Scientific, USA). Band densities were quantified using a ChemiDoc XRS+ gel documentation system with Image Lab Software (Bio-Rad, USA). The following amounts of sample were loaded per well to detect the proteins (the dilutions of sera containing polyclonal antibodies are indicated in parenthesis): *Salmonella* FlgE, 10 μL supernatant protein or 0.5 μL cell pellet samples (*S. enterica* FlgE antibodies, 1:10,000 dilution); *Salmonella* FliC, 5 μL supernatant protein or 0.5 μL cell pellet samples (*S. enterica* FliC antibodies, 1:20,000 dilution); *Campylobacter* FlgE, 10 μL supernatant protein or 10 μL cell pellet samples (*C. jejuni* FlgE antibodies, 1:10,000 dilution); and *Campylobacter* flagellin, 2 μL supernatant protein or 2 μL cell pellet samples (*C. jejuni* FlaA antibodies, 1:5000 dilution). Sera from rabbits containing polyclonal antibodies reactive towards *S. enterica* FlgE or FliC were generously provided by T. Minamino (Osaka University). Sera from rabbits containing polyclonal antibodies reactive towards *C. jejuni* FlgE and FlaA proteins were made in this study.

### Electron microscopy

*Salmonella* strains were examined by electron microscopy similarly as described previously [51]. Cultures were grown in LB with antibiotics as required to late exponential phase (OD_600_ nm 1.0) at 37 °C. Cell suspensions were spotted on Mextaform HF-34 200-mesh carbon-coated copper grids and the cells were stained with 1% phosphotungstic acid at pH 7. Grids were examined using a JEM-1230R transmission electron microscope (JEOL, Ltd., Japan) at 100 kV. At least 30 negatively stained cells were examined for each strain. Flagella numbers per cell for each strain were compared using a Mann–Whitney *U* test. Box plots were prepared using BoxPlotR (http://shiny.chemgrid.org/boxplotr/).

*Campylobacter* strains were examined by electron microscopy similarly as described by Hendrixson and DiRita [48]. Briefly, the strains were grown on Mueller–Hinton agar, containing appropriate antibiotics, at 42 °C under microaerophilic conditions for 36 h. Cells were gently scraped from the surface of the plate using a sterile plastic inoculating loop and stained with phosphotungstic acid as described above for *Salmonella*.

## Additional files

Additional file 1: Molecular modeling calculations on the flagellar rod/hook intrinsically disordered segment. (A) Plot of energy vs. RMSD to the reference structure for the ID-Rod-Stretch of FlgE of *C. jejuni* (3500 structures), FlgE of *S. enterica* (2000 structures), and FliC of *S. enterica* (1000 structures). Models (A) are generated by Rosetta, blue and red lines show the median energy ± twice the median absolute deviation. Only low energy structures are shown in the main plot; an inset with the same units shows the distribution for all models, with those in the main plot highlighted in green. Energies are given in Rosetta energy units (REU). Structure distributions showing the flexibility of the ID-Rod-Stretch, in the case of FlgE from *S. enterica* (B) and FliC from *S. enterica* (C). Structures shown are a random sample of the Rosetta models colored according to their ranking from blue (low energy) to red (high energy). Ranges for the colour scales are 100 to 500 REU (B) or –60 to 40 REU (C). (D) Histograms showing the distribution of energies from the full set of Rosetta models for all three structures under consideration. The disordered nature of the ID-Rod-Stretch sequences is apparent from the lack of any well-separated population of low energy structures, in contrast with similar results from *S. enterica* FliC; the absence of any favored structure is particularly the case for the large *C. jejuni* ID-Rod-Stretch. (JPG 3805 kb)

Additional file 2: Comparison of the flagellar rod intrinsically disordered segment in *Campylobacter* and in *Salmonella*. (A) View of the linker connecting domains D0 and D1 of the hook protein FlgE in *C. jejuni*. The linker is colored in cyan. (B) Rainbow colored (from blue to red) view of the ID-Rod-Stretch connecting domains D0 and D1 in FlgE of *C. jejuni* (left, cryo-electron microscopy structure) and *S. enterica* (right, theoretical model). (C) Sequence alignment of the N-terminal region of FlgE from *S. enterica* and of *C. jejuni*. The ID-Rod-Stretch is in blue for *S. enterica* and red for *C. jejuni*. The four conserved residues in FlgE family are highlighted in yellow. The alignment is performed assuming that the N-terminal methionine is cleaved. (JPG 3170 kb)

Additional file 4: Normal flagellar biosynthesis for a *Salmonella flgE* null mutant strain harboring a plasmid carrying the *flgE* gene. (A) Motility in soft tryptone agar of the wild-type strain (SJW1103), which harbored empty plasmid vector and a Δ*flgE* null mutant strain, which harbored a plasmid carrying the wild-type *flgE* gene. The plate was incubated for 6 h at 30 °C. Western blot analysis of FlgE (B) and FliC (C) proteins exported into the culture supernatant broth for cells grown to early stationary phase in LB at 37 °C. FliC is 51 kDa and FlgE is 42 kDa. Relative band densities (%) compared to those of wild-type are labelled. Box plot (D) displaying the distribution of the numbers of flagella per cell, for > 30 cells, and typical electron micrograph images of the wild-type strain (E) or the Δ*flgE* mutant strain that harbored a plasmid carrying the *flgE* gene (F). Bars, 1 μm. (JPG 4745 kb)

Additional file 5: Flagellar phenotypes for *S. enterica* producing FlgE ID-Rod-Stretch mutant proteins. (DOCX 41 kb)

Additional file 6: *Salmonella* expressing FlgE ID-Rod-Stretch mutant proteins. Electron micrographs of representative cells synthesizing FlgE proteins: (A) FlgE wild-type, (B) AAAAA(37–41), (C) AAAAA(42–46), (D) AAAAA(47–51), (E) AAAAA(52–56), and (F) AAAAA(62–66). Bars, 1 μm. Cells synthesizing FlgE AAAAA(27–31), AAAAA(32–36), or AAAAA(57–61) were aflagellate and are not shown. Histograms display the number of cells (*y* axis) with numbers of flagella per cell (*x* axis). Means and standard deviations for *N* cells are shown. Asterisks indicate that flagella numbers were significantly (*P* < 0.05) different compared to cells producing FlgE wild-type, analyzed using a two-tailed Mann–Whitney *U* test. (JPG 5827 kb)

Additional file 7: Schematic distribution of FlgE molecules in the hook. The distal rod (FlgG), the hook (FlgE), and the filament (FliC) are helical structures characterized by rotation and rise along their axis, resulting in structures consisting of 11 protofilaments marked here by letters A to K. Each rectangle represents a molecule of FlgE (in the case of the hook). The numbers indicate the sequential order of the molecules as the structure is built. Yellow arrows indicate some of the interactions with close neighbors. (JPG 5849 kb)

Additional file 8: Strains of bacteria and plasmids. (DOCX 44 kb)

Additional file 9: Oligonucleotide primers. (DOCX 45 kb)
