# Supplementary figures and images for: An intrinsically disordered linker controlling the formation and the stability of the bacterial flagellar hook
Source: BMC Biol. 2017 Oct 27;15:97. doi: 10.1186/s12915-017-0438-7 (PMC5660449; doi:10.1186/s12915-017-0438-7)

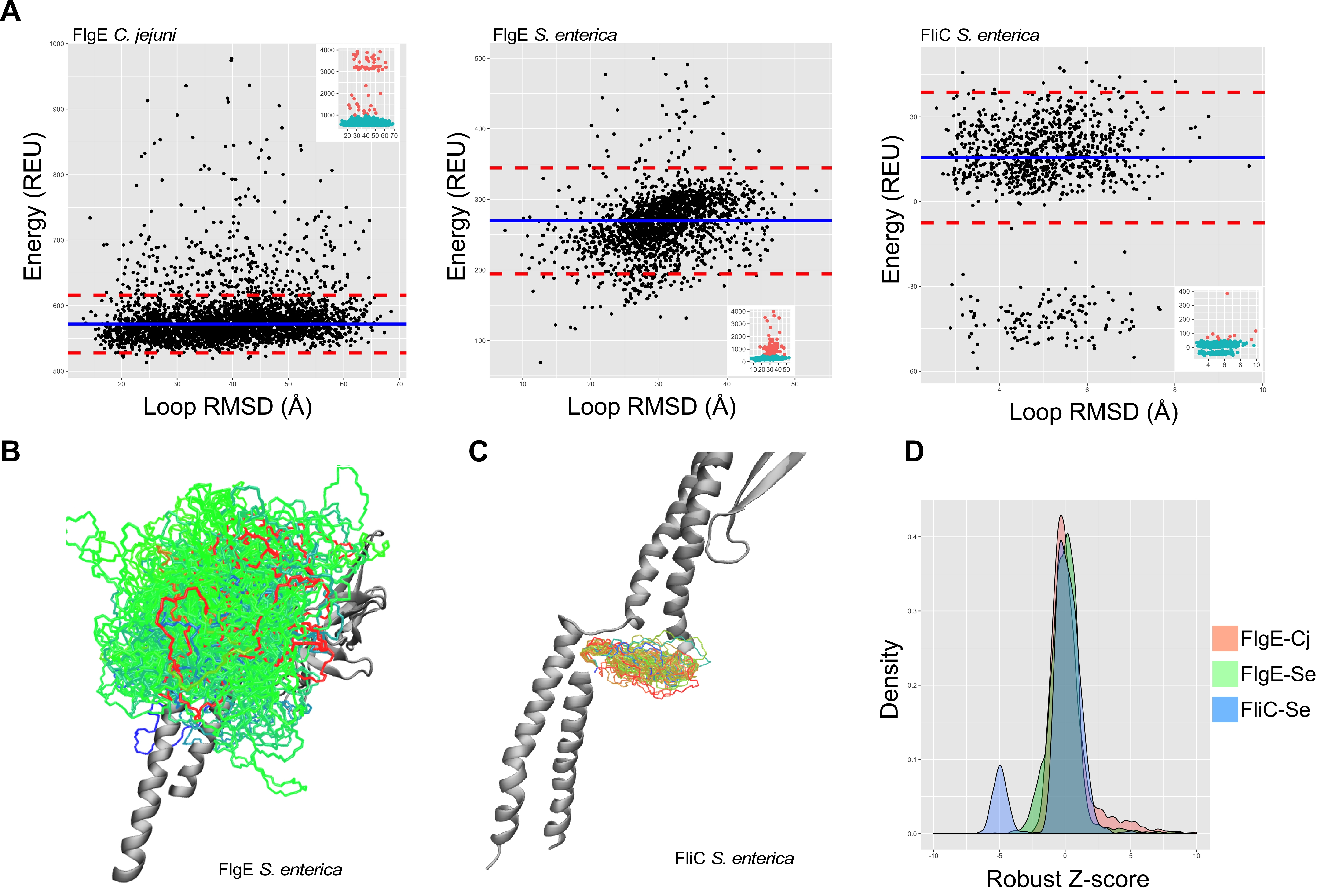

Supplement: Supplementary file 2 — Molecular modeling calculations on the flagellar rod/hook intrinsically disordered segment. (A) Plot of energy vs. RMSD to the reference structure for the ID-Rod-Stretch of FlgE of C. jejuni (3500 structures), FlgE of S. enterica (2000 structures), and FliC of S. enterica (1000 structures). Models (A) are generated by Rosetta, blue and red lines show the median energy ± twice the median absolute deviation. Only low energy structures are shown in the main plot; an inset with the same units shows the distribution for all models, with those in the main plot highlighted in green. Energies are given in Rosetta energy units (REU). Structure distributions showing the flexibility of the ID-Rod-Stretch, in the case of FlgE from S. enterica (B) and FliC from S. enterica (C). Structures shown are a random sample of the Rosetta models colored according to their ranking from blue (low energy) to red (high energy). Ranges for the colour scales are 100 to 500 REU (B) or –60 to 40 REU (C). (D) Histograms showing the distribution of energies from the full set of Rosetta models for all three structures under consideration. The disordered nature of the ID-Rod-Stretch sequences is apparent from the lack of any well-separated population of low energy structures, in contrast with similar results from S. enterica FliC; the absence of any favored structure is particularly the case for the large C. jejuni ID-Rod-Stretch. (JPG 3805 kb) [file 12915_2017_438_MOESM1_ESM.jpg]

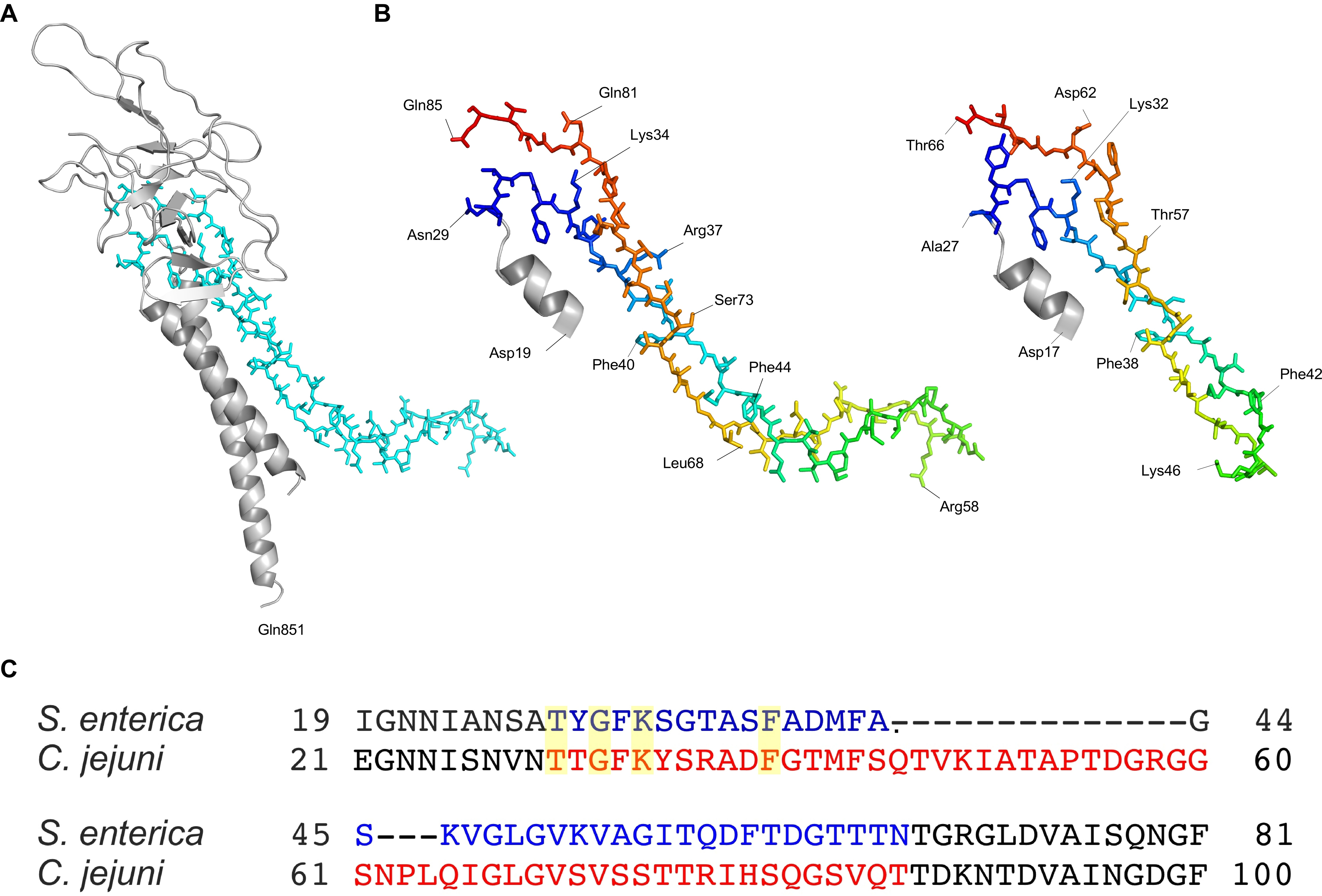

Supplement: Supplementary file 3 — Comparison of the flagellar rod intrinsically disordered segment in Campylobacter and in Salmonella. (A) View of the linker connecting domains D0 and D1 of the hook protein FlgE in C. jejuni. The linker is colored in cyan. (B) Rainbow colored (from blue to red) view of the ID-Rod-Stretch connecting domains D0 and D1 in FlgE of C. jejuni (left, cryo-electron microscopy structure) and S. enterica (right, theoretical model). (C) Sequence alignment of the N-terminal region of FlgE from S. enterica and of C. jejuni. The ID-Rod-Stretch is in blue for S. enterica and red for C. jejuni. The four conserved residues in FlgE family are highlighted in yellow. The alignment is performed assuming that the N-terminal methionine is cleaved. (JPG 3170 kb) [file 12915_2017_438_MOESM2_ESM.jpg]

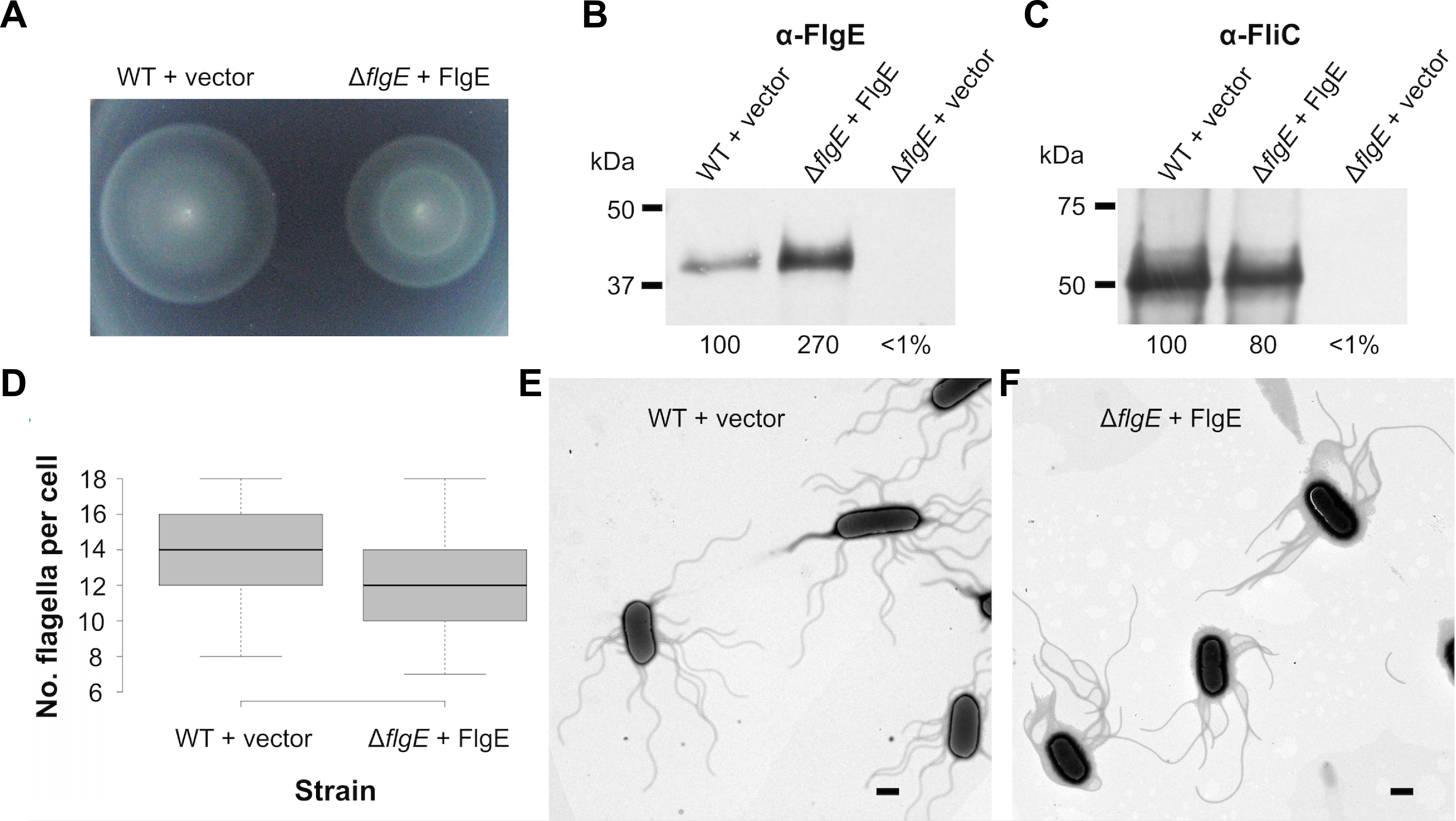

Supplement: Supplementary file 4 — Normal flagellar biosynthesis for a Salmonella flgE null mutant strain harboring a plasmid carrying the flgE gene. (A) Motility in soft tryptone agar of the wild-type strain (SJW1103), which harbored empty plasmid vector and a ΔflgE null mutant strain, which harbored a plasmid carrying the wild-type flgE gene. The plate was incubated for 6 h at 30 °C. Western blot analysis of FlgE (B) and FliC (C) proteins exported into the culture supernatant broth for cells grown to early stationary phase in LB at 37 °C. FliC is 51 kDa and FlgE is 42 kDa. Relative band densities (%) compared to those of wild-type are labelled. Box plot (D) displaying the distribution of the numbers of flagella per cell, for > 30 cells, and typical electron micrograph images of the wild-type strain (E) or the ΔflgE mutant strain that harbored a plasmid carrying the flgE gene (F). Bars, 1 μm. (JPG 4745 kb) [file 12915_2017_438_MOESM4_ESM.jpg]

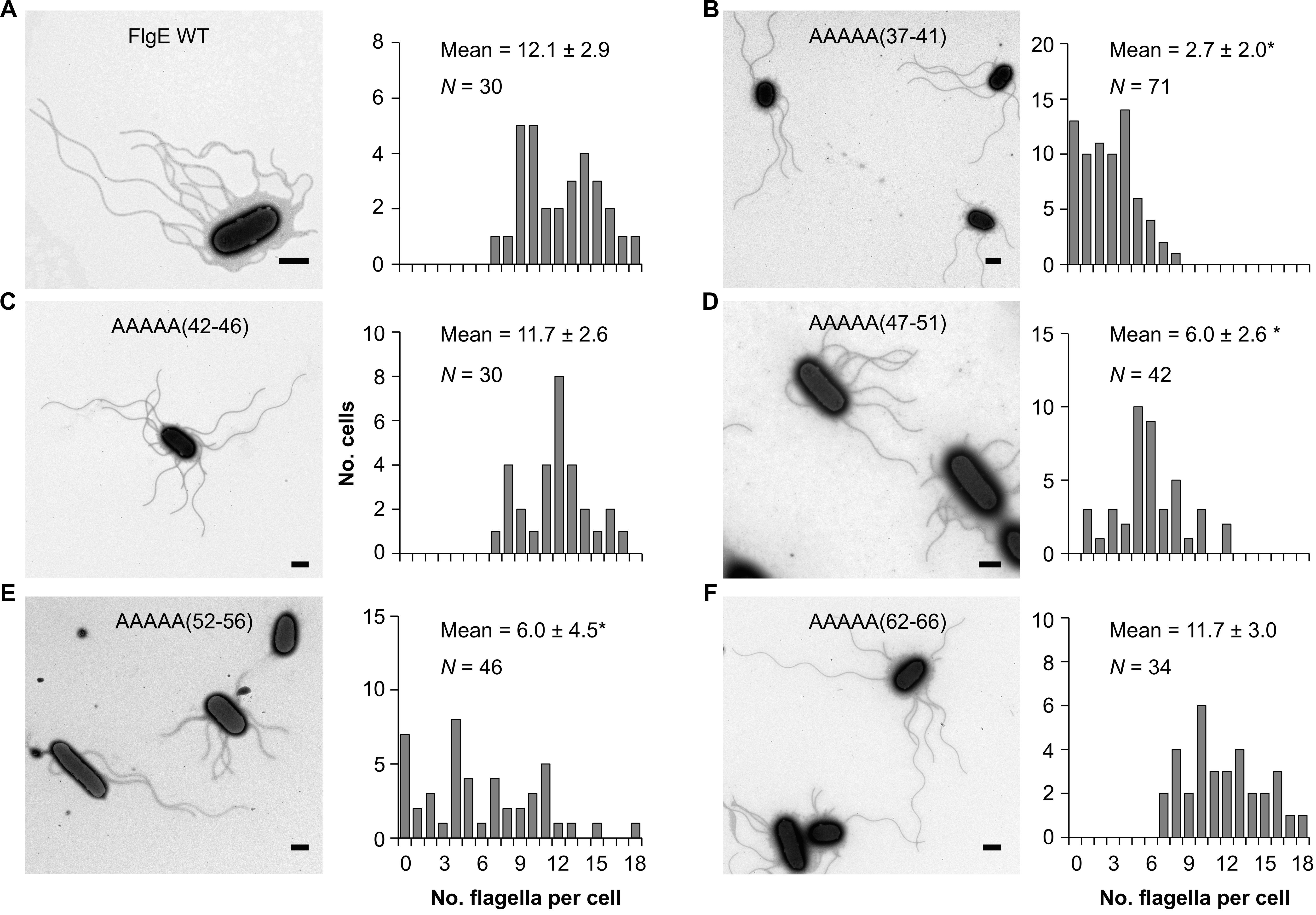

Supplement: Supplementary file 6 — Salmonella expressing FlgE ID-Rod-Stretch mutant proteins. Electron micrographs of representative cells synthesizing FlgE proteins: (A) FlgE wild-type, (B) AAAAA(37–41), (C) AAAAA(42–46), (D) AAAAA(47–51), (E) AAAAA(52–56), and (F) AAAAA(62–66). Bars, 1 μm. Cells synthesizing FlgE AAAAA(27–31), AAAAA(32–36), or AAAAA(57–61) were aflagellate and are not shown. Histograms display the number of cells (y axis) with numbers of flagella per cell (x axis). Means and standard deviations for N cells are shown. Asterisks indicate that flagella numbers were significantly (P < 0.05) different compared to cells producing FlgE wild-type, analyzed using a two-tailed Mann–Whitney U test. (JPG 5827 kb) [file 12915_2017_438_MOESM6_ESM.jpg]

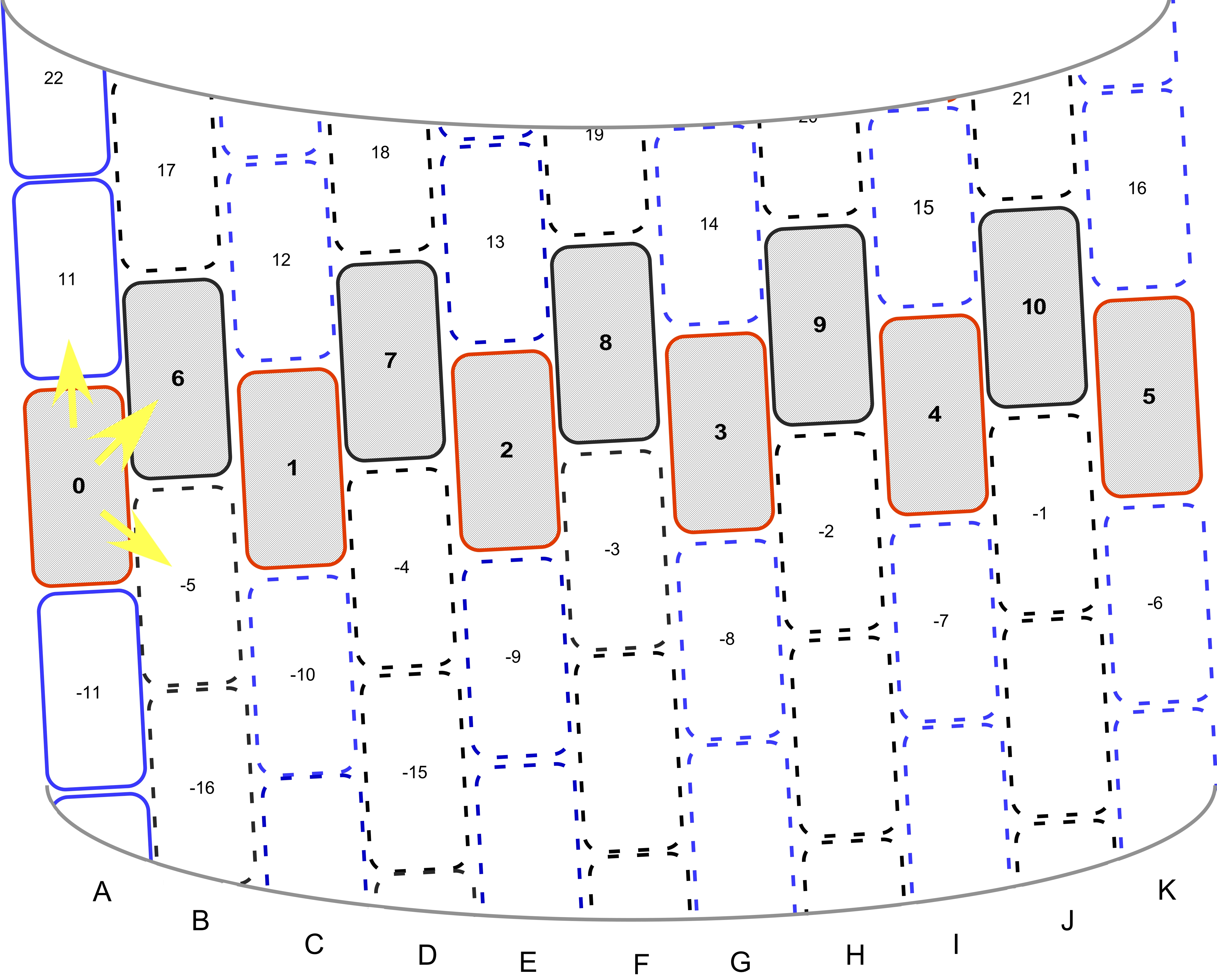

Supplement: Supplementary file 7 — Schematic distribution of FlgE molecules in the hook. The distal rod (FlgG), the hook (FlgE), and the filament (FliC) are helical structures characterized by rotation and rise along their axis, resulting in structures consisting of 11 protofilaments marked here by letters A to K. Each rectangle represents a molecule of FlgE (in the case of the hook). The numbers indicate the sequential order of the molecules as the structure is built. Yellow arrows indicate some of the interactions with close neighbors. (JPG 5849 kb) [file 12915_2017_438_MOESM7_ESM.jpg]
