# Supplementary material for: An intrinsically disordered linker controlling the formation and the stability of the bacterial flagellar hook
Source: BMC Biol. 2017 Oct 27;15:97. doi: 10.1186/s12915-017-0438-7 (PMC5660449; doi:10.1186/s12915-017-0438-7)
Supplement: Supplementary file 5 — Flagellar phenotypes for S. enterica producing FlgE ID-Rod-Stretch mutant proteins. (DOCX 41 kb) [file 12915_2017_438_MOESM5_ESM.docx]

**Additional file 5.** Flagellar phenotypes for *S. enterica* producing FlgE ID-Rod-Stretch mutant proteins

| FlgE protein*^a^* | Motility phenotype  (% of FlgE WT)*^b^* | FlgE export  (% of FlgE WT)*^c^* | FliC export  (% of FlgE WT)*^c^* | No. flagella per cell*^d^* |
| --- | --- | --- | --- | --- |
| FlgE WT | 100 | 100 | 100 | 12.1 ± 2.9 |
|  |  |  |  |  |
| **Exchange of amino acids by alanine residues** | | | | |
| T28A, Y29A, G30A, F31A | <1 | 170 | 8 | <0.1* |
| K32A, S33A, G34A, T35A | 11 ± 2 | 180 | 10 | 0.1 ± 0.2* |
| S37A, F38A, D40A, M41A | 10 ± 3 | 40 | 40 | 2.7 ± 2.0* |
| F42A, G44A, S45A, K46A | 93 ± 3 | 90 | 90 | 11.7 ± 2.6 |
| V47A, G48A, L49A, G50A, V51A | 19 ± 2 | 130 | 190 | 6.0 ± 2.6* |
| K52A, V53A, G55A, I56A | 54 ± 3 | 200 | 90 | 6.0 ± 4.5* |
| T57A, Q58A, D59A, F60A, T61A | 4 ± 1 | 150 | 20 | <0.1* |
| D62A, G63A, T64A, T65A, T66A | 65 ± 4 | 70 | 160 | 11.7 ± 3.0 |
| K32A | 17 ± 1 | 230 | 7 | 0.1 ± 0.4* |
| K32A D62Y | 41 ± 5 | 220 | 40 | 0.6 ± 0.6* |
| T28A, G30A, F31A, K32A, F38A, M41A | <1 | 190 | 3 | <0.1 |

*^a^* An *S. enterica* Δ*flgE* null mutant strain harboured a plasmid carrying a gene encoding the wild-type FlgE protein or a FlgE mutant protein. The amino acids are counted assuming N-terminal methionine cleavage.

*^b^* Mean average and standard deviations of the swim ring diameter of cells expressing the FlgE protein as a percentage of the swim ring diameter of cells expressing the wild-type FlgE protein after 6 h incubation in soft-tryptone agar at 30 ^o^C. Each motility plate was repeated at least four times.

*^c^* Band densities of FlgE and FliC proteins detected using Western blotting.

*^d^* Mean average and standard deviations of the numbers of flagella per cell, for samples >30 cells. Asterisks indicate where numbers of flagella per cell were significantly (*P* < 0.05) different from FlgE WT. Data were analyzed with a two-tailed Mann-Whitney *U* test.

See also Figures 4, 5 and 6 in the main text, which relate to the data in this table.
