# Supplementary material for: An intrinsically disordered linker controlling the formation and the stability of the bacterial flagellar hook
Source: BMC Biol. 2017 Oct 27;15:97. doi: 10.1186/s12915-017-0438-7 (PMC5660449; doi:10.1186/s12915-017-0438-7)
Supplement: Supplementary file 8 — Strains of bacteria and plasmids. (DOCX 44 kb) [file 12915_2017_438_MOESM8_ESM.docx]

**Additional file 8. Strains of bacteria and plasmids**

| Strain | Genotype or description^a^ | Reference or source^b^ |
| --- | --- | --- |
| ***Escherichia coli*** | | |
| XL10-Gold | K-12 strain derivative | Agilent, USA |
| ***Salmonella enterica* serovar Typhimurium** | | |
| SJW1103 | *S. enterica* serovar Typhimurium LT2, phase-1 stable derivative; wild type for motility and chemotaxis | (36) |
| JR501 | Restriction-deficient, modification-proficient for converting *E. coli* plasmids to *Salmonella* compatibility | (37) |
| SJW1368 | ∆(*cheW-flhD*); non-motile and without flagella (Fla-) | (38) |
| CB-A44 | Δ*flgE22590*::FKF; Km^R^ |  |
| CB-A45 | Δ*flgE22590*::FRT |  |
| ***Campylobacter jejuni*** | | |
| *C. jejuni* 81116 | Genetically stable, motile strain | (39) |
| CB897 | Δ(*flaA-flaB*)::Km^R^ |  |
| CB991 | Δ*flgE*::Km^R^ | (19) |
| CB-A9 | Δ*flgE*::Km^R^ 16S rRNA-*flgE*-Apr^R^-tRNAala | (19) |
| CB-A137 | Δ*flgE*::Km^R^ 16S rRNA-*flgE*(*1-46*::*68-853*)-Apr^R^-tRNAala |  |
| **Plasmids** | | |
| pUC19 | High copy number plasmid in *E. coli*; Amp^R^ | Thermo Fisher Scientific, USA |
| pKD13 | PCR template for isolation of an FRT-flanked kanamycin resistance cassette; Amp^R^ and Km^R^ | (40) |
| pKD46 | Low-copy number plasmid carrying bacteriophage lambda Red *γ*, *β*, and *exo* genes; temperature-sensitive *ori*; Amp^R^ | (40) |
| pCP20 | Thermal induction of FLP synthesis; temperature-sensitive *ori*; Amp^R^ and Cam^R^ | (41) |
| pTrc99A-FF4 | Modified pTrc expression vector; Amp^R^ | (42) |
| pCB954 | pTrc99A-FF4 carrying *S. enterica* *flgE* |  |
| pCB-A62 | pTrc99A-FF4 carrying *S. enterica* *flgE*(*T29A*) |  |
| pCB-A63 | pTrc99A-FF4 carrying *S. enterica* *flgE*(*G31A*) |  |
| pCB-A64 | pTrc99A-FF4 carrying *S. enterica* *flgE*(*F32A*) |  |
| pCB-A65 | pTrc99A-FF4 carrying *S. enterica* *flgE*(*K33A*) |  |
| pCB-A66 | pTrc99A-FF4 carrying *S. enterica* *flgE*(*F39A*) |  |
| pCB-A67 | pTrc99A-FF4 carrying *S. enterica* *flgE*(*M42A*) |  |
| pCB-A82 | pTrc99A-FF4 carrying *S. enterica* *flgE*(*T29A*, *Y30A*, *G31A*, *F32A*) |  |
| pCB-A83 | pTrc99A-FF4 carrying *S. enterica* *flgE*(*K33A*, *S34A*, *G35A*, *T36A*) |  |
| pCB-A84 | pTrc99A-FF4 carrying *S. enterica* *flgE*(*S38A*, *F39A*, *D41A*, *M42A*) |  |
| pCB-A85 | pTrc99A-FF4 carrying *S. enterica* *flgE*(*F43A*, *G45A*, *S46A*, *K47A*) |  |
| pCB-A86 | pTrc99A-FF4 carrying *S. enterica* *flgE*(*V48A*, *G49A*, *L50A*, *G51A*, *V52A*) |  |
| pCB-A87 | pTrc99A-FF4 carrying *S. enterica* *flgE*(*K53A*, *V54A*, *G56A*, *I57A*) |  |
| pCB-A88 | pTrc99A-FF4 carrying *S. enterica* *flgE*(*T58A*, *Q59A*, *D60A*, *F61A*, *T62A*) |  |
| pCB-A89 | pTrc99A-FF4 carrying *S. enterica* *flgE*(*D63A*, *G64A*, *T65A*, *T66A*, *T67A*) |  |
| pCB-A90 | pTrc99A-FF4 carrying *S. enterica* *flgE*(*T29A*, *G31A*, *F32A*, *K33A*) |  |
| pCB-A91 | pTrc99A-FF4 carrying *S. enterica* *flgE*(*T29A*, *G31A*, *F32A*, *K33A*, *F39A*, *M42A*) |  |
| pCB-A147 | pTrc99A-FF4 carrying *S. enterica* *flgE*(*K33A*, *D63Y*) |  |
| pCB956 | pUC19 carrying *C. jejuni* 81116 16S rRNA-*flgE*-Apr^R^-tRNAala-tRNAile-28S rRNA | (19) |
| pCB-A128 | pUC19 carrying *C. jejuni* 81116 16S rRNA-*flgE*(*1-46*::*68-853*)-Apr^R^-tRNAala-tRNAile-28S rRNA |  |

^a^ Gene numbering of amino acid residues refers to the codon position.

^b^ All bacterial strains and plasmids were made in this study, unless otherwise indicated.
