# Supplementary material for: An intrinsically disordered linker controlling the formation and the stability of the bacterial flagellar hook
Source: BMC Biol. 2017 Oct 27;15:97. doi: 10.1186/s12915-017-0438-7 (PMC5660449; doi:10.1186/s12915-017-0438-7)
Supplement: Supplementary file 9 — Oligonucleotide primers. (DOCX 45 kb) [file 12915_2017_438_MOESM9_ESM.docx]

**Additional file 9. Oligonucleotide primers**

| Name^a^ | Sequence (5` to 3`) | Strain or plasmid |
| --- | --- | --- |
| Fd-flgESe-FKF | taagcccttacacttatcaggagtcagtcatgtctttttctcaaattccggggatccgtcgacc | CB-A44 |
| Rv-flgESe-FKF | agctatcccgtcaggcgcttagcgcaggttaaccagcgtattgagtgtaggctggagctgcttc | CB-A44 |
| Fd-NdeI-flgESe | ggaaacag**catatg**tctttttctcaagcggttag | pCB954 |
| Rv-BamHI-flgESe | ctctaga**ggatcc**ttagcgcaggttaaccag | pCB954 |
| Fd-flgESe(T29A) | atcgccaactccgccgcctatggctttaagtc | pCB-A62 |
| Rv-flgESe(T29A) | gacttaaagccataggcggcggagttggcgat | pCB-A62 |
| Fd-flgESe(G31A) | actccgccacctatgcctttaagtccggtac | pCB-A63 |
| Rv-flgESe(G31A) | gtaccggacttaaaggcataggtggcggagt | pCB-A63 |
| Fd-flgESe(F32A) | tccgccacctatggcgctaagtccggtacggc | pCB-A64 |
| Rv-flgESe(F32A) | gccgtaccggacttagcgccataggtggcgga | pCB-A64 |
| Fd-flgESe(K33A) | actccgccacctatggctttgcgtccggtacgg | pCB-A65 |
| Rv-flgESe(K33A) | ccgtaccggacgcaaagccataggtggcggagt | pCB-A65 |
| Fd-flgESe(F39A) | ggctttaagtccggtacggcatcagctgccgatatgttcgc | pCB-A66 |
| Rv-flgESe(F39A) | gcgaacatatcggcagctgatgccgtaccggacttaaagcc | pCB-A66 |
| Fd-flgESe(M42A) | cggcatcatttgccgatgcgttcgccggttccaaag | pCB-A67 |
| Rv-flgESe(M42A) | ctttggaaccggcgaacgcatcggcaaatgatgccg | pCB-A67 |
| Fd-flgESe(T29A-Y30A-G31A-F32A) | gtaataacatcgccaactccgccgccgctgccgctaagtccggtacggcatcatttg | pCB-A82 |
| Rv-flgESe(T29A-Y30A-G31A-F32A) | caaatgatgccgtaccggacttagcggcagcggcggcggagttggcgatgttattac | pCB-A82 |
| Fd-flgESe(K33A-S34A-G35A-T36A) | gccaactccgccacctatggctttgcggccgctgcggcatcatttgcc | pCB-A83 |
| Rv-flgESe(K33A-S34A-G35A-T36A) | ggcaaatgatgccgcagcggccgcaaagccataggtggcggagttggc | pCB-A83 |
| Fd-flgESe(S38A-F39A-D41A-M42A) | gctttaagtccggtacggcagcagctgccgctgcgttcgccggttccaaagtgg | pCB-A84 |
| Rv-flgESe(S38A-F39A-D41A-M42A) | ccactttggaaccggcgaacgcagcggcagctgctgccgtaccggacttaaagc | pCB-A84 |
| Fd-flgESe(F43A-G45A-S46A-K47A) | tacggcatcatttgccgatatggccgccgctgccgcagtggggctgggcg | pCB-A85 |
| Rv-flgESe(F43A-G45A-S46A-K47A) | cgcccagccccactgcggcagcggcggccatatcggcaaatgatgccgta | pCB-A85 |
| Fd-flgESe(V48A-G49A-L50A-G51A-V52A) | tgttcgccggttccaaagcggcggcggccgcaaaagtggcggggattac | pCB-A86 |
| Rv-flgESe(V48A-G49A-L50A-G51A-V52A) | gtaatccccgccacttttgcggccgccgccgctttggaaccggcgaaca | pCB-A86 |
| Fd-flgESe(K53A-V54A-G56A-I57A) | ggttccaaagtggggctgggcgtagcagcggcggcggctacccaggatttt | pCB-A87 |
| Rv-flgESe(K53A-V54A-G56A-I57A) | aaaatcctgggtagccgccgccgctgctacgcccagccccactttggaacc | pCB-A87 |
| Fd-flgESe(T58A-Q59A-D60A-F61A-T62A) | ggggctgggcgtaaaagtggcggggattgccgcggctgctgccgacggtacgacaa | pCB-A88 |
| Rv-flgESe(T58A-Q59A-D60A-F61A-T62A) | ttgtcgtaccgtcggcagcagccgcggcaatccccgccacttttacgcccagcccc | pCB-A88 |
| Fd-flgESe(D63A-G64A-T65A-T66A-T67A) | acccaggattttaccgccgctgcggcagcgaacaccgggcgcgg | pCB-A89 |
| Rv-flgESe(D63A-G64A-T65A-T66A-T67A) | ccgcgcccggtgttcgctgccgcagcggcggtaaaatcctgggt | pCB-A89 |
| Fd-flgESe(T29A-G31A-F32A-K33A) | aataacatcgccaactccgccgcctatgccgctgcgtccggtacggcatcatttgc | pCB-A90 |
| Rv-flgESe(T29A-G31A-F32A-K33A) | gcaaatgatgccgtaccggacgcagcggcataggcggcggagttggcgatgttatt | pCB-A90 |
| Fd-flgESe(F39A-M42A) | gtccggtacggcatcagctgccgatgcgttcgccggttccaaag | pCB-A91 |
| Rv-flgESe(F39A-M42A) | ctttggaaccggcgaacgcatcggcagctgatgccgtaccggac | pCB-A91 |
| Fd-flgECj(1-46::68-853) | gattttgggactatgtttagcggtcttggtgtttcagtaagt | pCB-A128 |
| Rv-flgECj(1-46::68-853) | acttactgaaacaccaagaccgctaaacatagtcccaaaatc | pCB-A128 |

^a^Numbering of amino acids refers to the codon position.
